# The Incidence of End-Stage Renal Disease in the Diabetic (Compared to the Non-Diabetic) Population: A Systematic Review

**DOI:** 10.1371/journal.pone.0147329

**Published:** 2016-01-26

**Authors:** Maria Narres, Heiner Claessen, Sigrid Droste, Tatjana Kvitkina, Michael Koch, Oliver Kuss, Andrea Icks

**Affiliations:** 1 Institute for Biometrics and Epidemiology, German Diabetes Center, Düsseldorf, Germany; 2 Department of Public Health, Heinrich-Heine-University, Düsseldorf, Germany; 3 Center of Nephrology, Mettmann, Germany; 4 Clinic of Nephrology, Heinrich-Heine-University, Düsseldorf, Germany; 5 German Center for Diabetes Research (DZD), München-Neuherberg, Germany; Baker IDI Heart and Diabetes Institute, AUSTRALIA

## Abstract

End-stage renal disease (ESRD) in diabetes is a life threatening complication resulting in a poor prognosis for patients as well as high medical costs. The aims of this systematic review were (1) to evaluate the incidence of ESRD due to all causes and due to diabetic nephropathy in the diabetic population and differences between incidences of ESRD with respect to sex, ethnicity, age and regions, (2) to compare incidence rates in the diabetic and non-diabetic population, and (3) to investigate time trends. The systematic review was conducted according to the PRISMA group guidelines by performing systematic literature searches in the biomedical databases until January 3^rd^ 2015; thirty-two studies were included. Among patients with incident type 1 diabetes the 30-year cumulative incidence ranged from 3.3% to 7.8%. Among patients with prevalent diabetes, incidence rates of ESRD due to all causes ranged from 132.0 to 167.0 per 100,000 person-years, whereas incidence rates of ESRD due to diabetic nephropathy varied from 38.4 to 804.0 per 100,000 person-years. The incidence of ESRD in the diabetic population was higher compared to the non-diabetic population, and relative risks varied from 6.2 in the white population to 62.0 among Native Americans. The results regarding time trends were inconsistent. The review conducted demonstrates the considerable variation of incidences of ESRD among the diabetic population. Consistent findings included an excess risk when comparing the diabetic to the non-diabetic population and ethnic differences. We recommend that newly designed studies should use standardized methods for the determination of ESRD and population at risk.

## Introduction

Diabetes mellitus (DM) has become a large worldwide public health problem. In recent years the global prevalence of diabetes mellitus has increased substantially, reaching 8.3% in 2014, which corresponds to 387 million patients [1]. In Western countries, diabetes is considered to frequently lead to chronic renal replacement therapy (RRT) due to ESRD. Published studies reported that among patients who started RRT the proportion of patients with diabetes ranged from 24% (only counting diabetic nephropathy) [2] to 51% (all causes counted) [3]. Taking into account the increasing prevalence of obesity [4–6] and diabetes [1] as well as improved survival patients with diabetes [7, 8] an increase of the incidence of ESRD can be expected with correspondingly severe individual and social consequences. ESRD is a life threatening complication resulting in a poor prognosis for individuals with DM. Epidemiological studies showed that the combination of ESRD and diabetes leads to an increased risk of cardiovascular events [9, 10]. A high incidence of cardiovascular disease leads to a shorter life expectancy in this group of patients. The published studies have demonstrated an increased mortality among ESRD patients with diabetes compared to ESRD patients without diabetes [3, 11–15]. Furthermore, the treatment of ESRD entails high medical costs [16–18]. Reducing diabetes-related ESRD by at least one third was declared as a 5-year goal in the St. Vincent Declaration in 1989 [19]. Since then, numerous epidemiological studies concerning the incidence of ESRD in the diabetic population have been conducted. The published results have demonstrated considerable variation in the incidence of ESRD, especially with respect to time trends [3, 20–22]. Moreover, the studies differed largely with respect to study population and study design, and hence they are difficult to compare. Few non-systematic reviews dealing with this topic have been performed, and most of these were published prior to 2000 [23, 24] or analyzed the incidence of ESRD among persons with diabetes in relation to the general population [25, 26]. No systematic review has been performed up to now. Given the lack of systematic knowledge, we have conducted the first systematic review concerning the incidence of ESRD in diabetic (compared to non-diabetic) patients.

The main objectives of this review were (1) to evaluate the incidence of ESRD due to all causes and due to diabetic nephropathy in the diabetic population and differences between incidences of ESRD with respect to sex, ethnicity, age and regions, (2) to compare incidence rates in the diabetic and non-diabetic population, and (3) to investigate time trends.

## Methods

The systematic review was performed according to the PRISMA (Preferred Reporting Items for Systematic Reviews and Meta-Analyses) guidelines [27] (S1 Table).

### Data Sources and Searches

A comprehensive literature search was conducted by searching the international biomedical literature databases MEDLINE, EMBASE, Web of Knowledge and publisher databases Journals@OVID and ScienceDirect in February 2013. The last update was performed on 3^rd^ January 2015. Systematic search strategies were developed and processed by using database-specific controlled terms (MeSH, EMTREE) and additional free text terms. The search terms (combined by Boolean operators) were diabetic nephropathy, nephritis, nephrosclerosis, glomerulonephritis, end-stage renal disease, renal replacement therapy, hemodialysis, renal dialysis, peritoneal dialysis, kidney transplantation etc. to compose the “ESRD” search component (PICO), and epidemiology, prevalence, incidence, frequency, population survey, survey data, administrative data etc. to compose the “epidemiology” search component. The systematic search is based on a linear block building model. Some berry picking strategies were added. Moreover, we used an additional handsearch to search potential eligible studies as reference lists of review articles and relevant studies. The detailed search strategies are provided in the S1 Text.

### Study Selection

#### Inclusion criteria

Full-text articles were included if they met inclusion criteria with respect to types of studies, types of population and the main outcome regardless of time period of study and year of publication, definition of ESRD, type of diabetes, age, sex and ethnicity.

Types of population: The population at risk had to be (1) defined by official statistics, which means for example all inhabitants of a defined region or all persons with statutory health insurance, and (2) individuals where diabetes (incident or prevalent) should be known or anticipated. Hence, the population at risk in the study could be (i) all incident individuals with diabetes within a defined population, or (ii) all prevalent individuals with diabetes within a defined population, or (iii) the general population, divided into those with and those without diabetes. Individuals with diabetes could have type 1 diabetes, type 2 diabetes or a specification of the type of diabetes was not performed. We also considered other diabetes classifications, namely insulin dependent (IDDM) und non-insulin dependent diabetes mellitus (NIDDM). Individuals without diabetes were also considered with the aim of comparing incidences between the diabetic and non-diabetic population.

Outcomes: ESRD definition, epidemiological measures. Different definitions of ESRD were considered: renal replacement therapy (RRT) including first dialysis or a transplantation due to chronic kidney disease; first dialysis; overall dialysis (dialysis before and after transplantation); combination of RRT or dialysis with death due to ESRD; ESRD diagnosis based exclusively on clinical symptoms and/or laboratory analyses. Furthermore, ESRD was analyzed as ESRD due to all causes among patients with diabetes or exclusively ESRD due to diabetic nephropathy. The main outcome was the incidence of ESRD among patients with DM considering the incidence rate (IR) or cumulative incidence (CumI) of ESRD. In order to compare incidence rates of ESRD between the diabetic and non-diabetic population, the relative risks (RR) were also taken into account.

Types of studies: All population-based longitudinal studies using both a prospective and retrospective (quasi prospective) design were included in this review.

#### Exclusion criteria

Studies were excluded if they were published in a language other than English. We also excluded studies if they solely reported incidences of ESRD among persons with DM in relation to the total population (diabetic and non-diabetic) and not exclusively to the diabetic (possibly compared to the non-diabetic) population.

### Data collection and extraction

Two authors (H.C. and A.I.) independently screened all the retrieved titles and abstracts identified through the search strategies to identify potentially eligible articles. Subsequent full-text screening and data extraction was performed by two authors (M.N. and H.C.). Disagreements were resolved by discussion with a third reviewer (A.I.). Data extraction was performed including information about first author, publication year, country, study period, study design, study population, type of diabetes, definition of ESRD, cause of ESRD and data sources of the study. With regard to the results, the number of cases of ESRD, type of incidence measure (CumI or IR, crude or adjusted) for the total population at risk and stratified by sex and ethnic origin (if available), as well as time trends (if available), were extracted. The reported IR was recalculated as IR per 100,000 PY in the diabetic population, if not originally reported as such.

### Quality Assessment

In the final selection of eligible studies we assessed features that could potentially bias the estimates of ESRD using the Cochrane approach Study Quality Guide [28]. These included the measuring of the outcome ESRD (how ESRD was defined or recorded), the reporting of outcome measures (IR, CumI and RR), estimates of incidence and RR (given with confidence intervals and age-sex-adjusted), as well as the analysis and reporting of the time trends (descriptive or based on multivariate regression models). Using this tool we defined criteria based on clinical and epidemiological expertise and ranked potential sources of bias into low or high risk of bias according to the recommendations of the Cochrane approach Study Quality Guide (Table 1).

**Table 1:** Characteristics considered for assessment of risk of bias adapted to Cochrane approach Study Quality Guide [28].

Assessment items	Lower risk of bias	Higher risk of bias
Definition of outcome ESRD	Precise definition and description of how the ESRD was recorded	No definition and description of how the ESRD was recorded
Diagnostic criteria of diabetes	Documented by physician (clinical diagnosis, ICD)	Self-reported DM
Statistical methods: IR, CumI, RR	Presented as age-sex-adjusted estimates; reported with CI	Crude rates; reported without CI
Time trends	Time trends reported using multivariate regression model	Time trends reported only descriptively
Duration of the observation period^a^	5 years and more	Less than 5 years

CI, confidence interval; CumI, cumulative incidence; DM, diabetes mellitus; ICD, International Statistical Classification of Diseases and Related Health Problems; IR, incidence rate; RR, relative risk.

^a^ Relevant for studies reporting time trend.

### Categorization of studies

Due to the high heterogeneity of the studies, we analyzed the outcomes of interest in accordance with the reported diabetic population (incident or prevalent cases), cause of ESRD (all causes or only due to DN), type of diabetes, and incidence measures (IR or CumI). The following categories resulted: (a) studies describing incidence of ESRD due to DN in population with incident diabetes, (b) studies describing incidence of ESRD due to all causes in population with incident diabetes, (c) studies describing incidence of ESRD due to DN in population with prevalent diabetes, and (d) studies describing incidence of ESRD due to all causes in population with prevalent diabetes. In each group, we further classified the results by incidence measures (CumI or IR), type of diabetes, and definition of ESRD (e.g. RRT or dialysis) as well as crude and adjusted estimates. We decided not to show the incidence rates in cohorts with incident diabetes due to the difficulty of interpreting these results. We therefore only presented the cumulative incidence in populations with incident diabetes. The results from studies reporting relative risks were presented in a separate table.

### Statistic methods

The results (IR, CumI, and RR) from included studies were presented as age-sex-adjusted estimates if available, otherwise crude rates were reported. All estimates were presented with 95% confidence intervals (95% CI) if available. In either case, the number of events of ESRD was included in the results tables. We described time trend as “descriptive” if the reviewed studies have reported only annual incidence ESRD without using any multivariate regression models.

From previous experience, we expected that the studies would be too heterogeneous to allow for a quantitative summary of results. Indeed, and as judged with regard to content and not by performing prior statistical tests for homogeneity we found all estimates too heterogeneous and performed no meta-analyses.

## Results

### Searching and inclusion

**Fig 1:**
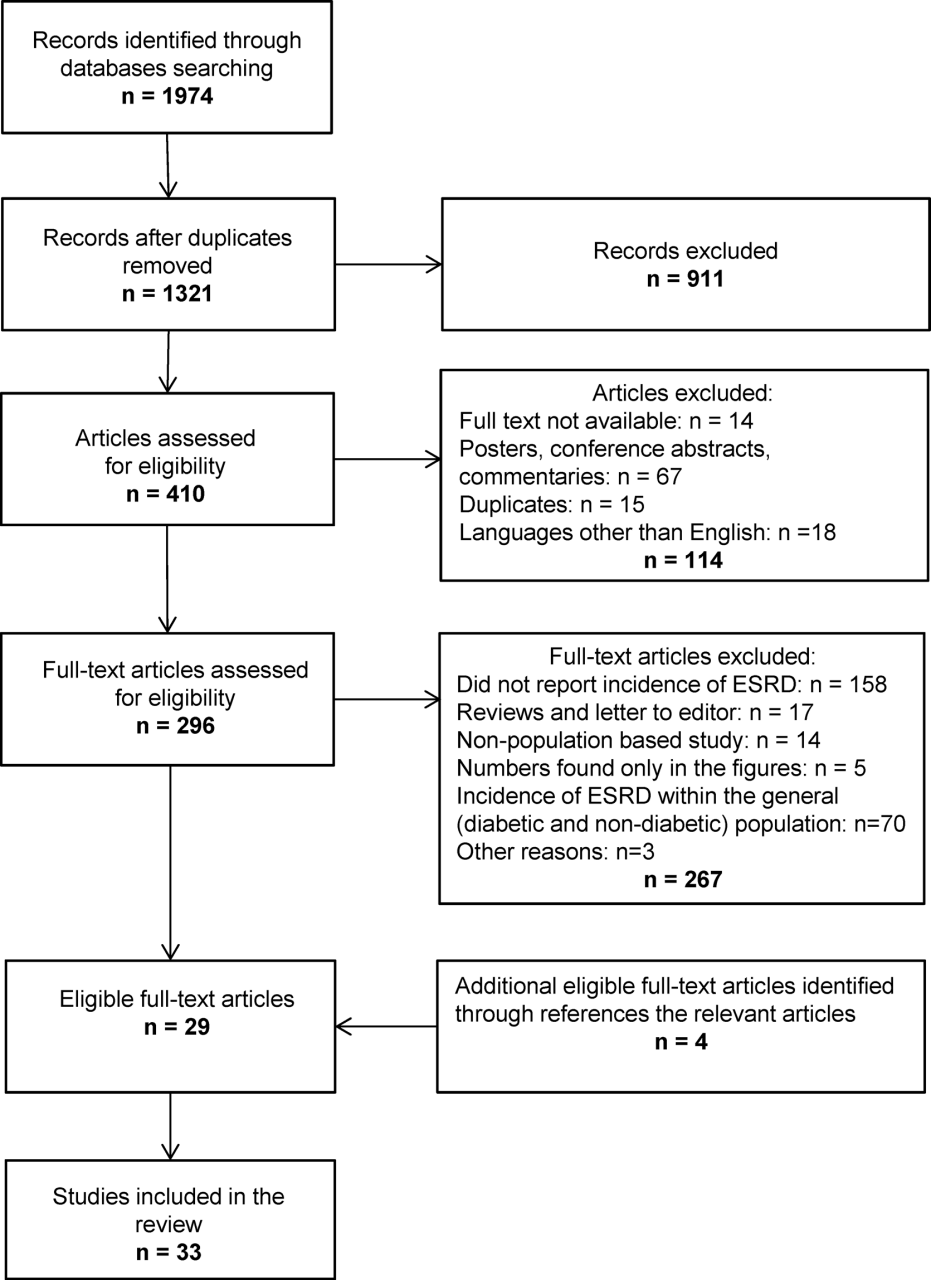
Flowchart of the systematic review process.

Initially we retrieved 1,974 citations, from which 29 papers met our inclusion criteria. Four additional articles were identified by checking the reference lists of selected papers. Hence, 33 articles were included in this review. The selection procedure is presented in Fig 1.

### Study design and quality assessment

With regard to the main outcome, different definitions of ESRD were considered. Most studies (n = 24) used RRT, three studies RRT or dialysis in combination with death due to ESRD, two studies first dialysis, three studies overall dialysis (n = 3), and one study ESRD based on clinical symptoms and/or laboratory analyses (Table 2). The majority of studies that reported incidence of ESRD did not differentiate the diabetes type (n = 18), followed by studies solely analyzing type 1 diabetes (n = 8) or type 2 diabetes (n = 1 as type 2 diabetes, n = 3 as NIDDM), and studies investigating both types of diabetes but differentiating between NIDDM and IDDM (n = 3).

**Table 2:** Incidence of ESRD in populations with incident and prevalent diabetes–study characteristics.

Study, year of publication, country	Study period, study design	Population characteristics, number at risk (n)	Definition of diabetes	Definition of ESRD	Data sources	Time trend	Estimates
**a. Incidence of ESRD due to diabetic nephropathy in the population with incident diabetes**							
***I*. *Cumulative incidence***							
***1*.*Type 1 diabetes***							
Möllsten et al. 2010 Sweden[37]	1991–2007 (all incident cases of DM were recorded for age 0–14 years since 1977 and for age 15–34 years since 1983) Prospective	All incident cases of T1DM in Sweden with DD at least 13 years. Age at onset 0–14 and 15–34 years n = 11,681	T1DM	RRT	Swedish Childhood Diabetes Registry, Diabetes Incidence prospective cohort Study (population) Swedish renal registry (cases)		CumI with death as competing risk
**b. Incidence of ESRD due to all causes in the population with incident diabetes**							
***I*. *Cumulative incidence***							
***1*. *Type 1 diabetes***							
Finne et al. 2005 Finland[38]	1965–1999 Follow-up to 2001 Prospective	All patients with T1DM in Finland. Age at onset DM < 30 years n = 20,005	T1DM	RRT	Finnish diabetes registry (population) Finish registry of kidney disease (cases)	+	CumI
Nishimura et al. 2003 USA[39]	1965–1979 Follow-up: 1999 Prospective	All patients with T1DM in Allegheny County. Age at onset DM < 18 years n = 798	T1DM	RRT	Population-based diabetes incidence registry (population) Questioning of the patients (cases)	+	CumI
Lin et al. 2014 Taiwan[40]	1999–2010 Retrospective	All patients with T1DM in Taiwan n = 7203	T1DM	First Dialysis	Taiwan National Health Insurance Research Database (both for diabetic population and cases)	+	CumI
***2*. *Type 2 diabetes/NIDDM***							
Humphrey et al. 1989 USA[29]	1945–1979 Follow-up to 1984 Retrospective	Patients with NIDDM from Rochester diabetic incidence cohort n = 1832	NIDDM^a^	Chronic renal failure defined by laboratory results: a) Creatinine ≥ 4.0 mg/dl; b) Urea ≥ 150 mg/dl if Creatinine < 4.0 mg/dl	Rochester diabetic incidence retrospective cohort study (population), Follow-up examinations including medical records (cases)	+	CumI
***3*. *Without differentiating the types of diabetes***							
Dyck et al. 2014 Canada[41]	1980–2005 Retrospective	All patients with DM in Saskatchewan. Age at onset DM <20 years n = 2640	Without differentiating	RRT	Saskatchewan´s universal healthcare system (both for diabetic population and cases)	-	CumI
**c. Incidence of ESRD due to diabetic nephropathy in the population with prevalent diabetes**							
***I*. *Cumulative incidence*: *No studies were found***							
***II*. *Incidence rates***							
***1*. *Type 1 diabetes***							
Matsushima et al.1995 Japan/USA[42]	Japan: two nationwide surveys (1970, 1981) USA: 1965–1979 Follow-up: 1990 Prospective	Patients with T1DM aged ≤18 years at onset DM between 1965 and 1979 two cohorts from Japan n = 1279 USA n = 794	T1DM	Overall dialysis	Two nationwide surveys (population Japan) Allegheny County IDDM Registry (population USA) Questionnaires to the physicians, telephone contacts (cases)	-	Diabetes duration-adj. IR
Uchigata et al. 2004 Japan[30]	1970–1981 Follow-up: 1990 Prospective	Persons with T1DM aged ≤18 years at onset DM between 1965 and 1979 n = 1374 DD was reported stratified by treatment center	T1DM	Overall dialysis and death due to ESRD	Two nationwide surveys (DERI Cohort) (population) Follow-up including questionnaires mailed to attending physicians or by telephone interview with patients or their families, in case of death detailed questionnaire of attending physicians (cases)	-	Crude IR
***2*. *Type 1 diabetes/IDDM//type 2 diabetes/NIDDM// both***							
Cowie CC et al. 1989 USA[43]	1974–1983 Retrospective	Michigan residents with DM aged ≥ 15 years at the onset of ESRD The analysis by type of DM: aged at the onset of ESRD 15–64 years n: NA	IDDM NIDDM both	RRT	1980 Michigan census (total population), National Health Interview Survey (diabetes prevalence) Michigan kidney registry (cases)	-	Age-sex-adj. IR
Pugh et al. 1995 USA[44]	1987–1991 Retrospective	Population with DM of region San Antonio and Dallas (Texas, USA) n: NA	IDDM NIDDM both	Overall dialysis	1990 census data (total population) Health and Nutrition Examination Survey (HANES) II and Hispanic HANES (diabetes prevalence) Population-based incidence cohort of dialysis center (cases)	-	Age-adj. IR
Stephens et al. 1990 USA[45]	1983–1984 Retrospective	Population with DM of region Kentucky und southwest Ohio n: NA	IDDM NIDDM both	RRT	1980 US census (total population) National Health Interview Surveys (diabetes prevalence) Patient population of network 17 (dialysis units that participate in the registry) (cases)	-	Crude IR
***3*. *Without differentiating the types of diabetes***							
Burrows et al. 2010 USA[20]	1990–2006 Retrospective	Estimated U.S. population with DM n: NA	All	RRT	Census (total population) National Health Interview survey (diabetes prevalence) US Renal Data System (cases)	+	Crude IR Age-adj. IR
Burrows et al. 2005 USA[46]	1990–2001 Retrospective	Southwestern American Indians (SWAI) with diabetes n: NA	All	RRT	Census (total population) Indian Health Service (IHS) (diabetes prevalence) US Renal Data System (cases)	+	Age-adj. IR
Burrows et al. 2014 USA[47]	1996–2010 Retrospective	Estimated Puerto Rican population with DM aged ≥ 18 years	All	RRT	Behavioral Risk Factor Surveillance System (diabetic population) US Renal Data System (cases)	+	Crude IR Age-adj. IR
CDC 2010 USA and Puerto Rico[48]	1996–2007 Retrospective	U.S. and Puerto Rican population with DM aged ≥ 18 years n: NA	All	RRT	Behavioral Risk Factor Surveillance System (diabetic population) US Renal Data System (cases)	+	Age-adj. IR
CDC 1992 USA[49]	1980–1989 Retrospective	U.S. population with DM n: NA	All	RRT	CDC's National Health Interview Survey (NHIS) (population) Medicare's ESRD program (cases)	+	Age-adj. IR
CDC 1992 USA[50]	1982–1989 Retrospective	Colorado residents with DM n: NA	All	RRT	Colorado population estimates (population) Intermountain End-Stage Renal Disease Network (Im ESRDN) (cases)	+	Age-adj. IR
Comas et al. 2012 Spain[36]	1994–2010 Retrospective	Catalonia residents with DM Hypertension: 1994–37%, 2010–59.6%; 1994 n = 243,120, 2010 n = 365,595	All	RRT	Catalonia Health Survey (population) Catalan renal registry (cases)	+	Crude IR Age-sex-adj. IR
Jones et al. 2005 USA[51]	1984–1996 Retrospective	U.S. population with DM 1984 n = 6.1 Mio.,1996 n = 8.5 Mio.	All	RRT	Diabetes Surveillance Program of the Centers for Disease Control and Prevention (population) US Renal Data System (cases)	+	Age-adj. IR
Lopes et al.1995 USA[52]	1988–1991 Retrospective	U.S. population with DM aged 20–79 years n: NA	All	RRT	US Bureau of the Census (total population) Second National Health and Nutrition Examination Survey (diabetes prevalence) US Renal Data System (cases)	-	Crude IR
Newman et al. 1990 USA[53]	1983–1986 Retrospective	American Indians with DM/ U.S. population with DM n = 72,000/4,892,000	All	RRT	1980 US Census (total population) Indian Health Service ambulatory care data, National figure for diabetic Whites (diabetes prevalence) Health Care Financing Administration's ESRD Medical Information System (cases)	-	Crude IR Age-adj. IR
Burden et al.1992 UK[54]	1979–1988 Retrospective	Leicestershire (UK), White and Asian population with DM ≥ 16 years n = NA	All	RRT	1981 OPCS census (total White pop), Leicester city survey (total Asian pop) Previously reported prevalence rates (source unknown) (diabetes prevalence) Department of Nephrology register of end-stage renal failure (cases)	-	Crude IR
Lorenzo et al. 2010 Spain[55]	2003–2006 Retrospective	Spanish population with DM aged > 16 years 2003 n = 1,887,041^†^; 2006 n = 2,050,499^†^	All	RRT	Census population figures (total population) Spanish National Health Survey (diabetes prevalence) Spanish national registries (cases)	+	Crude IR
Muntner et al. 2003 USA[35]	1978 and 1991 Retrospective	U.S. population with DM ≥ 30 years. Hypertension: 1978–55%, 1991–54%; 1978 n = 5.5 Mio.,1991 n = 9.6 Mio.	All	RRT	United States Census (population) Second and Third National Health and Nutrition Examination Surveys (diabetes prevalence) United States Renal Data System (cases)	+	Crude IR
Gregg at al. 2014 USA [22]	1990–2010 Retrospective	Estimated U.S. population with and without DM aged ≥ 20 years n diabetic population 1990 / 2010: 6.5 Mio / 20.7 Mio.	All	RRT	Census (total population) National Health Interview survey (diabetes prevalence) US Renal Data System (cases)	+	Age-adj. IR
**d. Incidence of ESRD due to all causes in the population with prevalent diabetes**							
***I*. *Cumulative incidence***							
***1*.*Type 1 diabetes***							
Thomas et al. 2011 Finland [31]	1998–2002 Follow-up: 2010 Prospective	All patients from the FinnDiane prospective cohort. Age at onset DM 15–34 years DD at baseline 20 years, HbA1c 8.4% Hypertension 47% Cholesterol total 5.0 mmol/l HDL 1.3 mmol/l n = 2807	T1DM	RRT	Nationwide multicenter study (FinnDiane) (population) Search in the renal registries and center databases and verified from medical files (cases)	-	Crude CumI
LeCaire et al. 2014 USA[32]	1980–1982 Follow-up: 2005–2007 Prospective	Patients from Wisconsin Epidemiologic Study of Diabetic Retinopathy (WESDR) cohort. Age at onset DM < 30 years DD at baseline 15 years. n = 996 HbA1c 10.1% (87 mmol/mol) SysBD/ DiaBD 125/79 mmHg	T1DM	RRT	Prospective cohort study: baseline examination (population) Self-reported (cases)	+	Crude CumI
***II*. *Incidence rates***							
***1*. *Type 1 diabetes*: *No studies were found***							
***2*. *Type 2 diabetes/NIDDM***							
Bruno et al. 2003 Italy[33]	1991–2001 Prospective	Patients with known T2DM in Casale Monferrato (Italy) DD at baseline 10.7 years HbA1c 8.4% Cholesterol total 5.79 mmol/l HDL 1.42 mmol/l n = 1565	T2DM	First dialysis	Prospective cohort study: baseline examination (population) follow-up examinations at diabetic clinic or general practitioners (cases)	-	Crude IR
Lee et al. 1994 USA[56]	1972–1980 Follow up: 1987–1990 Prospective	Oklahoma Indians with NIDDM DD 6.9 years n = 912	NIDDM	RRT and death due to ESRD	Prospective cohort study: baseline examination at the Indian Hospitals of the U.S. Public Health Service and their satellite clinics (population), follow-up examination including physical examination and laboratory tests, medical records of persons not further pursued (cases)	-	Crude IR
Nelson et al.1988 USA[34]	1975–1986 Retrospective	Pima and Papago Indians with NIDDM Age at onset DM ≥ 5 years n total = 5059 DD was presented stratified by age classes	NIDDM	Dialysis and death due to ESRD	Retrospective cohort study (population, diabetes prevalence) Register of patients undergoing chronic renal dialysis, patient registers of all dialysis centers in Phoenix area, Network 6 ESRD Registry, death certificates, autopsy findings, medical examiner’s reports (cases)	-	Crude IR RR
***3*. *Without differentiating the types of diabetes***							
Icks et. al 2011 Germany[21]	2002–2008 Retrospective	Population of one region in Germany age ≥ 30 years n total ∼ 310 000	All	RRT	Federal Office for Statistics (total population) East German diabetes register (diabetes prevalence) Data of regional dialysis center (cases)	**+**	Age-adj. IR Age-sex-adj. IR RR
Hoffmann et al. 2011 Germany[13]	2005–2006 Follow-up to 2008 Retrospective	All insured persons of one statutory health insurance company aged ≥ 30 years; n = 789,858	All	RRT	One statutory health insurance company in Germany (population, diabetes prevalence and cases)	-	Age-adj. IR Age-sex-adj. IR RR
Lok et al. 2004 Canada[3]	1994–2000 Follow-up to 2001 Retrospective	Ontario residents aged ≥20 years n total = 8,405,626, n diabetic population = 528,874	All	Overall dialysis	Prospective population-based cohort study of two cohorts (with and without DM population) Ontario diabetes database (diabetes prevalence) Claims to the Ontario Health Insurance Plan (cases)	+	Crude IR Age-sex-adj. IR RR
Muntner et al. 2003 USA[35]	1991Retrospective	US Population Diabetic population USA ≥ 30 years in 1991 Diabetic population n = 9.6 Mio. Hypertension: 1978–55%, 1991–54%	All	RRT	United States Census (population) Second and Third National Health and Nutrition Examination Surveys (diabetes prevalence) United States Renal Data System (cases)	-	Crude IR RR

DD, diabetes duration; DM, diabetes mellitus; DN, diabetic nephropathy; CI, confidence interval; CumI, cumulative incidence; ESRD, end-stage renal disease; HR, hazard ratio; IDDM, insulin dependent diabetes mellitus; IR, incidence rate(s); NA, not available; NIDDM, non-insulin dependent diabetes mellitus; PY, person-years; RRT, renal replacement therapy; RR, relative risk; SysBD/ DiaBD, systolic blood pressure/diastolic blood pressure; T1DM, type 1 diabetes mellitus; T2DM, type 2 diabetes mellitus; Y, year.

^a^ Results in the population with IDDM are not presented due to low number of cases ESRD (N = 3).

† self-calculated.

All of the studies reviewed used an appropriate study design: nine studies were prospective and 25 were retrospective (quasi prospective). Most of the studies (n = 28) had sourced their data concerning ESRD cases from a centralized database such as the national renal registry. The majority of studies included a minimum of four cases. The partial results from one study were not included due to the small number of cases (n = 3) yielding wide confidence intervals [29]. The included studies used different sources to estimate the population at risk (persons with diabetes): 19 studies used data from national surveys, five studies data from national or local diabetes registries, three studies data came from national or local insurance companies, and four studies sourced data directly from physicians’ or hospital records. Two studies reported diabetes prevalence without referring to reliable sources (Table 2).

Only six studies [30–34] reported duration of diabetes and few studies reported clinical risk factors for the development of ESRD such as glucose concentration [31–33], blood pressure [29, 31, 32, 35, 36] and lipids [31, 33]. Also cardiovascular disease was only rarely considered [3, 21, 29, 32, 36], and no study analyzed an interaction between cardiovascular disease and diabetes in the incidence of ESRD.

With regard to statistical significance, a third of the studies (n = 13) reported results with a 95% confidence interval. More than half (n = 18) of all the included studies took time trends into consideration; however, only twelve of them described the time trend with appropriate statistical methods. Due to the high heterogeneity of the included studies no meta-analysis was performed

### Main findings

#### Incidence of ESRD due to diabetic nephropathy in the population with incident diabetes

The CumI of ESRD due to DN was analyzed only in one study (Table 2A, Table 3A) and reached 3.3% after 30 years diabetes duration (DD) [37].

**Table 3:** Incidence of ESRD in populations with incident and prevalent diabetes–results.

Study, year of publication, country	Number of cases ESRD	Incidence: CumI (%),IR per 100,000 PY (95% CI)–total population	Incidence: CumI (%), IR per 100,000 PY (95% CI)–stratified by sex and ethnic origin	Time trend
**a. Incidence of ESRD due to diabetic nephropathy in the population with incident diabetes**				
***I*. *Cumulative incidence***				
***1*.*Type 1 diabetes***				
Möllsten et al. 2010 Sweden[37]	N = 127	30 Y CumI: 3.3	Men: 20 Y CumI: 0.7 (0.5–1.0), 25 Y CumI: 2.6 (2.0–3.3), 30 Y CumI: 4.0 (3.0–5.2); Women: 20 Y CumI: 0.7 (0.5–1.0), 25 Y CumI: 1.4 (1.0–2.0), 30 Y CumI: 2.4 (1.6–3.5), Ethnic origin: NA	NA
**b. Incidence of ESRD due to all causes in the population with incident diabetes**				
***I*. *Cumulative incidence***				
***1*. *Type 1 diabetes***				
Finne et al. 2005 Finland[38]	N = 632	20 Y CumI: 2.2 (1.9–2.5), 30 Y CumI: 7.8 (7.1–8.5)	Men: 20 Y CumI: 2.1 (1.7–2.5), 30 Y CumI: 8.3 (7.3–9.3); Women: 20 Y: 2.2 (1.7–2.6), 30 Y: 7.8 (6.7–8.8); Ethnic origin: NA	Lower risk of ESRD for patients whose diabetes was diagnosed in more recent years (RR, 95% CI) 1965–1969 1.00, 1970–1974 0.78 (0.64–0.94), 1975–1979 0.72 (0.57–0.90), 1980–1999 0.47 (0.34–0.65)
Nishimura et al. 2003 USA[39]	N = 104	20 Y CumI: 1965–1969: 9.1,1970–1974: 4.7, 1975–1979: 3.6, 25 Y CumI: 11.3	Sex: NA; Ethnic origin: White: 20 Y CumI: 5.2, Black: 20 Y CumI: 21.9	Differences between three onset cohorts were statistically significant (p = 0.006)
Lin et al. 2014 Taiwan[40]	N = 226	NA	Men: 10 Y CumI: 5.6; Women:10 Y CumI: 5.9	Lower risk of ESRD for patients whose diabetes was diagnosed in more recent years (HR, 95% CI) 1999–2002 1.00, 2003–2006 0.646 (0.47–0.88), 2007–2010 0.43 (0.23–0.8)
***2*. *Type 2 diabetes/NIDDM***				
Humphrey et al. 1989 USA[29]	N = 25	10 Y CumI: 0.8, 25 Y CumI: 6.2	Sex: NA; Ethnic origin: NA	descriptive
***3*. *Without differentiating the types of diabetes***				
Dyck et al. 2014 Canada[41]	N = 28	NA	Non-First Nations: 25 Y CumI: 4.3, First Nations: 25 Y CumI: 12.3	NA
**c. Incidence of ESRD due to diabetic nephropathy in the population with prevalent diabetes**				
***I*. *Cumulative incidence*: *No studies were found***				
***II*. *Incidence rates***				
***1*. *Type 1 diabetes***				
Matsushima et al. 1995 Japan/USA[42]	Japan: N = 72 USA: N = 37	IR adj. by DD Japan: 564.9 (433.8–696.0), IR adj. by DD USA: 295.6 (200.1–391.0)	Sex: NA, Ethnic origin: NA	NA
Uchigata et al. 2004 Japan[30]	N = 81	Crude IR: 546.0 †	Sex: NA; Ethnic origin: NA	NA
***2*. *Type 1 diabetes/IDDM//type 2 diabetes/NIDDN// both***				
Cowie CC et al.1989 USA[43]	Michigan residents with DM aged ≥ 15 years: N = 1331.The analysis by type of DM: aged 15–64 years: N = 594	NA	**Both types** age-sex adj. IR: White: 50.2 (46.9–53.5),White men: 65.7 (60.0–71.3),White women: 38.3 (34.4–42.2), Black: 127.8 (115.7–139.8), Black men: 148.1 (127.4–168.7), Black women: 112.1 (97.9–126.3)**; IDDM**^a^ age-sex-adj IR: White: 35.3 (30.1–40.4), White men: 52.2 (42.8–61.6),White women: 21.2 (15.9–26.4), Black: 36.3 (27.1–45.4), Black men: 36.4 (23.8–49.0), Black women: 36.2 (23.1–49.3)**; NIDDM**^a^ age-sex-adj. IR: White: 25.1 (20.8–29.5), White men: 28.9 (22.2–35.7),White women: 22.0 (16.4–27.6), Black: 108.2 (93.5–123.0), Black men: 126.0 (100.8–151.2), Black women: 93.4 (76.5–110.3)	NA
Pugh et al.1995 USA[44]	N = 648	NA	Sex: NA; Ethnic origin: **Both types** age-adj. IR: White: 125.5 (83.8–167.2), Mexican:117.8 (87.8–147.8), Black: 193.7 (141.2–246.2); **IDDM**^a^ age-adj. IR: White: 84.0 (46.8–121.2), Mexican: 14.0 (0.0–28.0), Black: 38.0 (5.45–70.6); **NIDDM**^a^ age-adj. IR: White: 41.5 (22.6–60.4), Mexican: 103.8 (77.3–130.4), Black: 155.7 (114.5–196.9)	NA
Stephens et al. 1990 USA[45]	N = 466	**T1DM** crude IR: 491.7, **T2DM** crude IR: 70.6	**T1DM:** Men: 592.0, Women: 394.0,White: 431.3,White men: 504.7,White women: 358.8, Black: 1237.3, Black men: 1761.5, Black women: 795.5; **T2DM**: Men: 69.8,Women: 71.1,White: 46.5,White men: 46.1,White women: 46.7,Black: 224.4, Black men: 255, Black women: 207.5	NA
***3*. *Without differentiating the types of diabetes***				
Burrows et al. 2010 USA[20]	1990: N = 17,727 2006: N = 48,215	Crude IR 1990/1996/2006: 285.4/421.9/ 278.4, Age-adj. IR 1990/1996/2006: 299.0/343.2/197.7	Men: age-adj. IR 1990/2006 363.7/230.5, Women: age-adj. IR 1990/1996/2006 250.6/ 299.3/ 168.6, Black: age-adj. IR 1990/2006: 408.9/327.7, White: age-adj. IR 1990/1996/2006: 266.2/ 296.7/164.7, Hispanic: age-adj. IR 1997/2006: 306.7/254.3	APC for age-adj. IR (95% CI) 1990–1996: APC +1.1 (- 1.9 to 4.1) p = 0.45, 1996/2006: APC -3.9 (- 4.7 to—3.1) p<0,01, Men:1990/2006 APC -2.8 (- 3.6 to—1.9) p = 0.01; Women: 1990–1996: APC +2.4 (- 1.5 to 6.4) p = 0.20; 1996/2006: APC -4.3 (- 5.3 to- 3.3) p<0,01, Black: 1990–2006: APC -1.7 (- 2.9 to—0.6) p <0.0,White: 1990–1996: APC +1.3 (- 3.0 to 5.7) p = 0.53, 1996/2006: APC -5.0 (- 6.0 to -4.0) p<0,01, Hispanic: 1997–2006: APC -1.5 (- 3.3 to 0.3) p = 0.09
Burrows et al. 2005 USA[46]	1990: N = 154 2001: N = 320	Age-adj. IR 1993: 804, Age-adj. IR 2001: 558	Sex: NA, Ethnic origin: NA	1990–2001 leveled off (p = 0.13) after reaching high points in 1993 and 1996. 1993–2001 decline 31% (p<0,05)
Burrows et al. 2014 USA[47]	1996: N = 536 2010 N = 970	Crude IR 1996/2010: 193.5/267.9, Age-adj. IR 1996/2000/2010:152.8/230.8/203.1	Men: age-adj. IR 1996/2001/2010:171.9/371.3/279.8, Women: age-adj. IR 1996/2010: 130.7/138.3	APC for age-adj. IR (95% CI) 1996–2000: APC +12.4 (3.3–22.4) p = 0.01, 2000–2010: APC—2.3 (- 4.1 to—0.5) p = 0.02, Men: 1996–2001 APC + 13.4 (6.9–20.3) p<0.001, 2001–2010 APC—3.1 (- 5.7 to 0.5) p = 0.03, Women: 1996–2010: APC—0.6 (-2.6 to 1.5) p = 0.56
CDC 2010 USA and Puerto-Rico[48]	1996: N = 32,716 2007:N = 48,712	Age-adj. IR USA and Puerto Rico:1996: 304.5 (288.8–320.3), 2007: 199.1 (193.9–204.2), Age-adj. IR Puerto Rico1996: 152.7 (127.5–177.9), 2007: 196.3 (166.5–226.2)	Sex: NA; Ethnic origin: NA	1996–2007 USA and Puerto Rico together: decreased (p<0.001), only Puerto Rico: increased by 29% (p<0.001)
CDC 1992 USA[49]	1980: N = 2220 1989: N = 13,332	Age-adj. IR 1980: 38.4, Age-adj. IR 1989: 202.0	Age-adj. IR 1989, White men: 201.3, Black men: 284.6,White women: 150.8, Black women: 352.8	descriptive
CDC 1992 USA[50]	N = 874	Age-adj. IR 1982: 61, Age-adj. IR 1989: 216	NA	descriptive
Comas et al. 2012 Spain[36]	1994: N = 119 2002: N = 203 2006: N = 210 2010: N = 217	Crude IR1994: 48.95 (40.15–57.74), 2002: 65.89 (56.83–74.96), 2006: 59.57 (51.51–67.62), 2010: 59.36 (51.46–67.25); Age-sex-adj.IR 1994: 50.91 (48.46–53.37), 2002: 64.53 (62.16–66.90), 2006: 60.26 (58.14–62.39), 2010: 60.00 (57.84–62.16)	Sex: NA; Ethnic origin: NA	descriptive
Jones et al. 2005 USA[51]	1984: N = 6981, 1996: N = 31,647	NA	Sex: NA, White age-adj. IR 1996: 312.1, Black: age-adj. IR 1996: 590.3	Average annual increment of IR: Age group < 45 years, White men: 7.3, Black men: 15.9, White women: 2.8, Black women 20.9, Age group 45–64:White: 19.2 Black: 34.3, Age group 65–74, White: 25.6 Black: 50.1,Age group 74 +, White: 15.4 Black: 36.1
Lopes et al. 1995 USA[52]	**	**	**	NA
Newman 1990 USA[53]	American Indians with DM: N = 1075, U.S. population with DM N = 75,291		Sex: NA; American Indians: age-adj. IR: 207.3,White: age-adj. IR: 103.7 †	NA
Burden et al.1992 UK[54]	Asian N = 10 White N = 14	NA	Sex: NA; Asian: crude IR 48.66 (18.51–78.81), Whites: crude IR 3.56 (1.70–5.42)	NA
Lorenzo et al. 2010 Spain[55]	2003:N = 969, 2006: N = 1095	Crude IR Spanish mainland 2003: min 17.7 (Basque Country), max 98.5 (Asturias), Crude IR Spanish mainland 2006:min 20.9 (Basque Country), max 63.7 (La Rioja), Crude IR Canary Islands 2003: 121.0, 2006: 147.7	Sex: NA; Ethnic origin: NA	2006 compared with 2003 Spanish mainland RR 1.014 (0.929–1.106) p = 0.76, Canary Islands RR 3.88 (3.07–4.89) p<0.001
Muntner et al. 2003 USA[35]	1978: N = 1281, 1991:N = 18,218	Crude IR 1978: 23.3†, Crude IR 1991: 189.8 †	Sex: NA; Ethnic origin: NA	descriptive
Gregg et al. 2014 USA [22]	1990: N = 17,763; 1995: N = 29,259; 2000: N = 41,477; 2005: N = 46,917; 2010: N = 50197	Age-adj. IR 1990: 279 (257–300), 1995: 345 (319–371), 2000: 286 (276–297), 2005: 236 (228–246), 2010: 200 (191–209)	Men: Age-adj. IR 1990/2000/2010: 324 (278–371) / 309 (290–327) / 218 (203–232), Women: Age-adj. IR 1990/2000/2010: 247 (225–269)/ 268 (256–280)/ 182 (171–192), White: Age-adj. IR 1990/2000/2010: 244 (221–266)/ 246 (235–256)/ 160 (152–169), Black: Age-adj. IR 1990/2000/2010: 444 (367–521) 478 (442–513) 366 (333–399)	Percent change 1990–2010: - 28.3 (-34.6 to—21.6), p<0.001

APC, annual percent change; DD, diabetes duration; DM, diabetes mellitus; DN, diabetic nephropathy; CI, confidence interval; CumI, cumulative incidence; ESRD, end-stage renal disease; HR, hazard ratio; IDDM, insulin dependent diabetes mellitus; IR, incidence rate(s); NA, not available; NIDDM, non-insulin dependent diabetes mellitus; PY, person-years; RRT, renal replacement therapy; RR, relative risk; T1DM, type 1 diabetes mellitus; T2DM, type 2 diabetes mellitus; Y, year.

^a^ denominator was the total diabetic population.

† self-calculated.

**results were only available stratified by age classes.

#### Incidence of ESRD due to all causes in the population with incident diabetes

Five studies reported the incidence of ESRD due to all causes in populations with newly diagnosed DM (Table 2B).

In the Canadian study without distinction between the types of diabetes, the CumI after 25 years DD reached 12.3% among First Nations people compared to 4.3% in their non-First Nations counterparts [41].

Differences between sex, ethnic origin and age: Only one study was able to show a significant association between gender and risk of ESRD [29]. Ethnicity as a risk factor for ESRD was described in two studies [39, 41]. An early age at the onset of DM was shown as a protective factor for ESRD in five studies [37, 38, 40, 29, 41] (data not shown).

Time-trend: Four studies reported a lower risk of ESRD for patients whose diabetes was diagnosed in more recent years [38–40, 29].

#### Incidence of ESRD due to diabetic nephropathy in the population with prevalent diabetes

Nineteen studies reported the incidence rate of ESRD due to DN (Table 2C).

Cumulative incidence: No studies were found

Incidence rates: When considering solely IDDM/ type 1 diabetes, the crude IR reached 491.7 (95% CI na) per 100,000 PY in the USA [45] and 546.0 (95% CI na) per 100,000 PY in Japan [30] (Table 3C). Age-adjusted IRs in the type 1 diabetic population varied between 295.6 and 564.9 (95% CI na) per 100,000 PY [42]. Among individuals with NIDDM, the IR of ESRD in the whole population was analyzed solely in one US-study, which estimated a crude IR of 70.6 (95% CI na) per 100,000 PY [45].

Among those studies without a distinction between types of diabetes, the analysis of crude IRs showed a broad range of IRs of ESRD due to DN from 17.7 (95% CI na) (Basque country, Spain, 2003) [55] to 421.9 (95% CI na) (USA, 1996) [20] per 100,000 PY. The age-adjusted rates varied between 38.4 (95% CI na) per 100,000 PY in the US population (USA, 1980) [50] and 804.4 (95% CI na) per 100,000 PY among American Indians (USA, 1993) [46].

Differences between sex, ethnic origin, age and geographic region: The vast majority of studies have shown that men were more likely to have ESRD than to women [20, 22, 43, 47, 50, 51]. Most studies analyzing the impact of ethnicity in the IR of ESRD due to DN have reported that the incidence of ESRD was substantially higher among Black, Hispanic, American Indian and Asian individuals than white diabetic individuals [20, 22, 25, 43–46, 50, 51, 54]. With regard to age, the highest IRs of ESRD due to DN were found in the age group 65–74 years [20, 22, 35, 51, 55]. Some geographical differences were found between the studies reviewed. While a study from Spain reported an age-sex-adjusted IR in 2010 of 60.0 (95% CI 57.84–62.16) per 100,000 PY [36], in the USA the age-adjusted IR for the same time period was 200 (95% CI 191–209) per 100,000 PY [22]. One US study reported marked differences with no clear geographical pattern in the USA with IR ranging between 108.3 (95% CI na) per 100,000 PY in Maine and 450.0 (95% CI na) per 100,000 PY in Hawaii [48]. Likewise, in Spain the IR was considerably higher in the Canary Islands than on the Spanish mainland [55]. When solely considering patients with type 1 diabetes, a Japanese study found that the age-adjusted IR was considerably higher in Japan than in the USA [42].

Time trends: US studies have shown that after increasing growth during the 1980s and early 1990s [20, 35, 49, 51], the incidence of ESRD due to DN has significantly decreased since the mid-1990s up to 2006 by -3.9% (95% CI: -4.7 to -3.1 to) per year [20]. Between 2006 and 2010 [22] the incidence rates did not change considerably (197.7 (95% CI na) and 200 (95% CI 191–209) respectively). Likewise, compatible results—albeit at a later time—were seen in Europe, where a Spanish study showed an increment of age-adjusted incidence from 1994 to 2002 (50.9 and 64.53 per 100,000 PY, respectively) with a subsequent decline to 60.0 in 2010 [36]. A recent study has demonstrated the same trend in Puerto Rico with an increase of incidence from 1996 to 2000 and subsequent reduction in 2010 [47]. In contrast, a strong increase of incidence was observed even in the 2000s in the Canary Islands [55].

#### Incidence of ESRD due to all causes in the population with prevalent diabetes

Nine studies reported the incidence of ESRD in populations with prevalent diabetes due to all causes. Four studies reported the incidence of ESRD exclusively in the diabetic population, while five studies analyzed the incidence rate of ESRD in both the diabetic and the non-diabetic population (Table 2D).

Cumulative incidence: Among patients with type 1 diabetes with a DD at baseline of around 20 years, the CumI after 10 years follow-up reached 4.5% (95% CI na) [31], whereas among their counterparts with a DD at baseline of 15 years the CumI after 25years follow-up reached 14.2% (95% CI: 11.9–16.5) [32] (Table 4).

**Table 4:** Incidence of ESRD in populations with prevalent diabetes partly compared to non-diabetic populations–results.

Study, year of publication, country	Number of cases ESRD (in diabetic/non-diabetic population)	Incidence: CumI (%), IR per 100,000 PY (95% CI) in diabetic/non-diabetic population—total	Incidence: CumI (%), IR per 100,000 PY (95% CI) in diabetic/non-diabetic population—stratified by sex and ethnic origin	RR (95% CI)—total population	RR (95% CI)—stratified by sex and ethnic origin	Time trend
**c. Incidence of ESRD due to diabetic nephropathy in the population with prevalent diabetes**						
***3*. *Without differentiating the types of diabetes***						
Gregg et al. 2014 USA [22]	1990: N = 17,763, 1995: N = 29,259, 2000: N = 41,477, 2005: N = 46,917, 2010: N = 50197	Age-adj. IR 1990: 279 (257–300), 1995: 345 (319–371), 2000: 286 (276–297), 2005: 236 (228–246), 2010: 200 (191–209)	Men: Age-adj. IR 1990/2000/2010: 324 (278–371)/309 (290–327) /218 (203–232); Women: Age-adj. IR 1990/2000/2010: 247 (225–269)/268 (256–280)/182 (171–192); White: Age-adj. IR 1990/2000/2010: 244 (221–266)/246 (235–256)/160 (152–169); Black: Age-adj. IR 1990/2000/2010: 444 (367–521)/478 (442–513)/366 (333–399)	1990: 13.7 (12.6–14.9), 2000: 9.5 (9.2–9.9), 2010: 6.1 (5.7–6.3)	NA	Percent change 1990–2010: - 28.3 (-34.6 to—21.6), p<0.001
***d*. Incidence of ESRD due to all causes in the population with prevalent diabetes**						
***I*. *Cumulative incidence***						
***1*.*Type 1 diabetes***						
Thomas et al. 2011 Finland [31]	N = 126	10 Y follow-up, CumI: 4.5	Sex: NA; Ethnic origin: NA	NA	NA.	NA
LeCaire et al. 2014 USA[32]	N = 68	25 Y follow-up, CumI:14.2 (11.9–16.5)	25 Y CumI: Men: 17.9 (14.3–21.5), Women: 10.3 (7.4–13.2,), Ethnic origin: NA	NA	NA	Comparing 1922–1969 with 1970–1980 unadjusted HR 0.29 (95% CI 0.19–0.44), fully adjusted HR 0.89 (95% CI 0.55–1.45)
***II*. *Incidence rates***						
***1*. *Type 1 diabetes*: *No studies were found***						
***2*. *Type 2 diabetes/NIDDM***						
Bruno et al. 2003 Italy [33]	N = 10	Crude IR: 104.0 (56–194)	Sex: NA; Ethnic origin: NA	NA	NA	NA
Lee et al. 1994 USA[56]	N = 64	Crude IR: 690	Sex: NA; Ethnic origin: NA	NA	NA	NA
Nelson et al. 1988 USA[34]	N = 76/4	Crude IR: 937.6/13.5†	Sex: NA; Ethnic origin: NA	Age-sex-adj. RR: 62 (20–188)	Sex: NA; Ethnic origin: NA	NA
***3*. *Without differentiating the types of diabetes***						
Icks et al. 2011 Germany[21]	N = 270/274	Age-sex-adj. IR: 167 (125–208)/20 (18–23)	Men: age-adj. IR: 213.6 (159.5–267.8)/26.9 (22.5–31.3); Women: age-adj. IR: 130.2 (65.6–194.9)/16.4 (13.5–19.3); Ethnic origin: NA	Age-sex-adj. RR: 8.3 (6.3–10.9)	Age-adj. RR: Men: 7.9 (5.9–10.8); Women: 8.0 (4.7–13.5); Ethnic origin: NA	No statistically significant time trend was found
Hoffmann et al. 2011 Germany[13]	N = 254/369	Age-sex-adj. IR: 157.9 (124.2–191.5)/25.6 (22.6–28.6)	Men: age-adj. IR: 186,6 (147.6–225.7)/41.0 (35.6–46.5); Women: Age-adj. IR: 135.1 (79.8–190.4)/15.4 (11.8–19.0); Ethnic origin: NA	Age-sex-adj. RR: 6.2 (4.8–7.9)	Age-adj. RR Men: 4.6 (3.6–5.8); Women: 8.8 (5.5–14.0); Ethnic origin: NA	NA
Lok et al. 2004 Canada[3]	1994–1995 N = 448/741, 1999–2000 N = 823/797	Age-sex-adj. IR: 1994–1995: 134.4/10.8, 1999–2000: 132.9/11.0	Diabetic population Men: crude IR: 1999–2000 167.0; Women: crude IR: 1999–2000 144.0	RR 1999–2000: 12.0	NA	In diabetic population annual reduction of 0.1%, non-diabetic population increase of 0.5%
Muntner, et al. 2003 USA[35]	1991 N = 24,767/24,351	Crude IR: 1991 256,7/18.7 †	Sex: NA; Ethnic origin: NA	Crude RR: 13.7 †	Sex: NA; Ethnic origin: NA	NA

DM, diabetes mellitus; CI, confidence interval; CumI, cumulative incidence; ESRD, end-stage renal disease; IR, incidence rate(s); NA, not available; PY, person-years; RRT, renal replacement therapy; RR, relative risk; T1DM, type 1 diabetes mellitus; T2DM, type 2 diabetes mellitus; Y, year.

† self-calculated.

Incidence rates in the diabetic population partly compared to the non-diabetic population:Crude IRs of ESRD among individuals with type 2 diabetes/NIDDM were reported from 104 (95% CI 56–194) per 100,000 PY among patients from Italy [33] to 690 (95% CI na) [56] and 937.6 (95% CI na) per 100,000 PY among American Indians [34] (Table 4).

Three studies, which estimated age-sex-adjusted IRs of ESRD without differentiating between the types of diabetes, showed comparable results with respect to both study design and results, and ranged between 132.9 (95% CI na) and 167 (95% CI 125–208) per 100,000 PY [3, 13, 21]. A considerably higher IR was found in the study by Muntner et al. with 256.7 per 100,000 PY [35].

Differences between gender, ethnic origin and age: All studies which stratified results by sex showed higher IR of ESRD among male patients [3, 13, 21, 32]. A considerably higher incidence of ESRD was reported among American Indians [34, 56]. Several studies showed a consistently strong increment of incidence of ESRD with increased age [13, 21, 32, 34, 35, 56].

Relative risks: RRs for ESRD in the diabetic population in comparison to the non-diabetic population (Table 4) were by far the highest among American Indians [34] with 62.0 (95% CI 20–188) while among the other studies the age-sex-adjusted RRs ranged from 6.2 (95% CI 4.8–7.9)[13] to 12 (95% CI na) [3]. Two German studies showed that the relative risks between individuals with and without diabetes decreased with increasing age in both men and women [13, 21] (data not shown).

One study analyzed RRs for ESRD due to DN in the diabetic population in comparison to the non-diabetic population (Table 4) and found a reduction of RRs between 1990 and 2010 from 13.7 (95% CI 12.6–14.9) to 6.1 (95% CI 5.7–6.3). [22].

Time trends: In the study describing CumI among patients with prevalent type 1 DM, a later time period of diabetes diagnosis was initially found to be a risk factor for the development of ESRD. Nevertheless, this trend was no longer significant after full adjustment [32]. Time-trends of ESRD in populations with and without diabetes (without differentiating the types of diabetes) were reported in two studies with inconsistent results: in the Canadian study from Lok, the age-adjusted IR of ESRD reduced during the time period 1994–2000 annually by only 0.1% in the diabetic population, while in the non-diabetic population this rate increased by 0.5% [3]. No apparent secular trend was found in the German study covering seven years [21].

## Discussion

ESRD among patients with diabetes is a life-threatening disease with poor survival rates and it is associated with high healthcare expenditure. This review was performed to analyze the incidence of ESRD due to all causes and due to diabetic nephropathy. We specified explicit eligibility criteria, conducted comprehensive searches, and assessed risk of bias using criteria specific to this review.

### Risk of bias within studies

Selection bias regarding the study population was minimized through the restriction to population-based studies. At the same time we detected some sources for information bias. Firstly, patients with unknown diabetes might have been misclassified as non-diabetic patients. Secondly, most of the studies did not take into account newly diagnosed cases of DM during the observation period. However, since individuals with incident ESRD or RRT are assumed to be more likely diagnosed with diabetes than those without incident ESRD or RRT, including newly diagnosed cases of DM could also cause bias. Finally, among patients with diabetes it is not always easy to determine the primary kidney disease leading to ESRD. This is especially true in the case of patients with type 2 diabetes. The cause of ESRD could be diabetic nephropathy (diabetes per se) or another disease (diabetes as comorbidity). With respect to the statistical analysis, not all the studies reported adjusted estimates or indicated 95% confidence intervals. This could lead to a reduced validity of the results of the study.

### Risk of bias across studies

Due to the fact that only articles published in the English language were reviewed, publication (language) bias could not be ruled out. Although we searched five databases, we cannot guarantee that some related papers may not have been identified. However, we did check the reference lists of reviewed articles to identify relevant studies. The studies reviewed used different definitions of ESRD (see methods section) that could cause detection bias. We minimized it by grouping together studies with a similar definition of outcome.

### Main findings

We identified 33 studies reporting incidences of ESRD in the diabetic population. Comparisons between studies were limited due to different measures of incidence, definitions of ESRD, diabetes types, observation time periods as well as the heterogeneous demographic characteristics (age, gender, ethnic origin) of the studied individuals. Hence, no quantitative data synthesis was performed due to a high degree of heterogeneity of the included studies. Nevertheless, there are some patterns that can be described.

#### Gender differences

Several studies have reported higher IRs of ESRD [3, 13, 20–22, 29, 47, 49, 50] and CumI [29, 32] among men than in women in the white population. In contrast, two studies found that in the black population women are more likely to develop ESRD [49, 50]. The higher IR of ESRD among white men may be explained partly by a higher prevalence of hypertension among males [21]. Nevertheless, it has been shown that among older patients with type 2 diabetes who were starting RRT, mortality rates were higher among women than men (adjusted HR for death comparing women and men: 1.19 (95% CI 1.08–1.30)) (P< 0.0003)[12].

#### Ethnic differences

Studies which analyzed incidence of ESRD with regard to ethnic differences without differentiating the type of DM reported higher IRs among black [20, 22, 43–45, 49–52], Hispanic [20, 44, 49, 50], Asian [54], American Indian [34, 46] and First Nations people [41] than their white counterparts. Constant higher relative risks ranging between 2 and 4 were found among black diabetic patients, irrespective of age and gender. The increased IR of ESRD among black and American Indian populations might be explained by genetic predisposition, higher prevalence of hypertension, environmental factors such as smoking, diet, and poor access to healthcare due to low socio economic status (SES) [17, 25, 57, 58]. Furthermore, a higher prevalence of micro- and macro-albuminuria was found among Asian, black and Hispanic individuals than in white patients in the Pathways Study on individuals with DM and identical access to good quality primary healthcare [17]. Moreover, in the Southern Community Cohort Study of 86,000 participants with low SES suffering from diabetes and hypertension, a higher risk of ESRD was shown for black people (HR: 2.4 (95% CI 1.9–3.0)) than white individuals after adjustment for sex, income, smoking status and comorbidities [59]. Ethnic disparities in the IR of ESRD among whites and non-whites (Black, Hispanic, Asian and Native American) were described in the earlier reviews [23, 57, 58].

#### International and regional differences

A comparison of the included studies by country showed that among patients with DM the incidence of ESRD was higher in the USA than in Europe and Canada. On the one hand, these differences could be due to ethnic differences, since the proportion of black residents who are more likely to undergo ESRD is higher in the USA than in Europe. On the other hand, a striking difference was also found when comparing the IR of ESRD due to DN between Norway’s population and the white US population (referring to the general population) [60]. Therefore, other factors could contribute to the discrepancy in the IR of ESRD between USA und Europe. Although no data were available, it has been hypothesized that access to healthcare, especially nephrology and diabetic care, and management of ESRD risk factors (e. g. blood pressure and metabolic control, lifestyle factors such as smoking) could partly explain this difference [60]. Regional disparities within a country were found in both the USA [48] and Spain [55]. Moreover, a similar pattern was shown in a recent study from France [61], although this study analyzed IRs of ESRD within the general population. The different background population with regard to age, ethnicity, SES and regional differences in medical care may partly explain these disparities [61].

#### Relative risks between diabetic and non-diabetic populations

**Fig 2:**
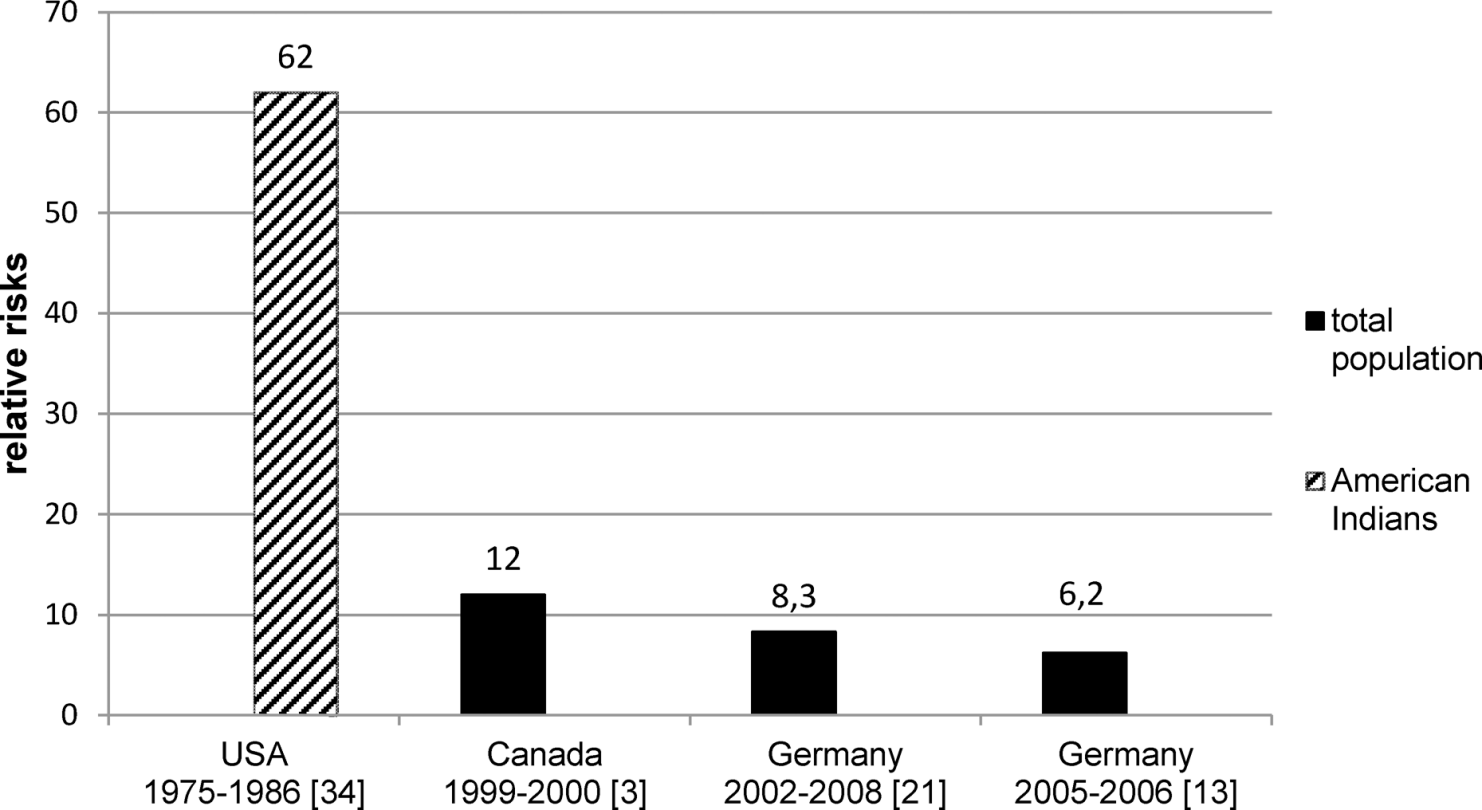
Age-sex-adjusted relative risks of ESRD due to all causes between diabetic and non-diabetic populations. ESRD, end-stage renal disease. Confidence intervals (CI) were not shown as not all studies reported incidence rates of ESRD with the corresponding 95% CIs.

As expected, all the studies included in this review have shown higher IRs of ESRD in the diabetic compared to the non-diabetic population. The age-sex-adjusted RRs for ESRD in the diabetic population in comparison to the non-diabetic population ranged between 6.2 [13] and 12 [3] in the general population [3, 13, 21] and reached 62 among Native Americans [34] (Fig 2). However, a comparison of these studies is difficult due to heterogenic study characteristic [22, 3, 13, 21, 34]. The two German studies [13, 21] analyzed a population aged 30 years and older while in the Canadian population [3, 22] individuals aged at least 20 years were included. The very high RR in the study by Nelson et al. could be explained by a low number of cases of ESRD among non-diabetic Native Americans (n = 4). One study estimated RRs for ESRD due to DN and found a decrease of these RRs between1990 and 2010 [22]. Results concerning gender-specific RR between diabetic and non-diabetic populations were inconsistent [13, 21]. A decreasing RR with increasing age [13, 21] could be explained by the strong rise in IRs of ESRD due to other reasons among older non-diabetic patients when compared to diabetic patients from the same age group.

#### Secular trend

**Fig 3:**
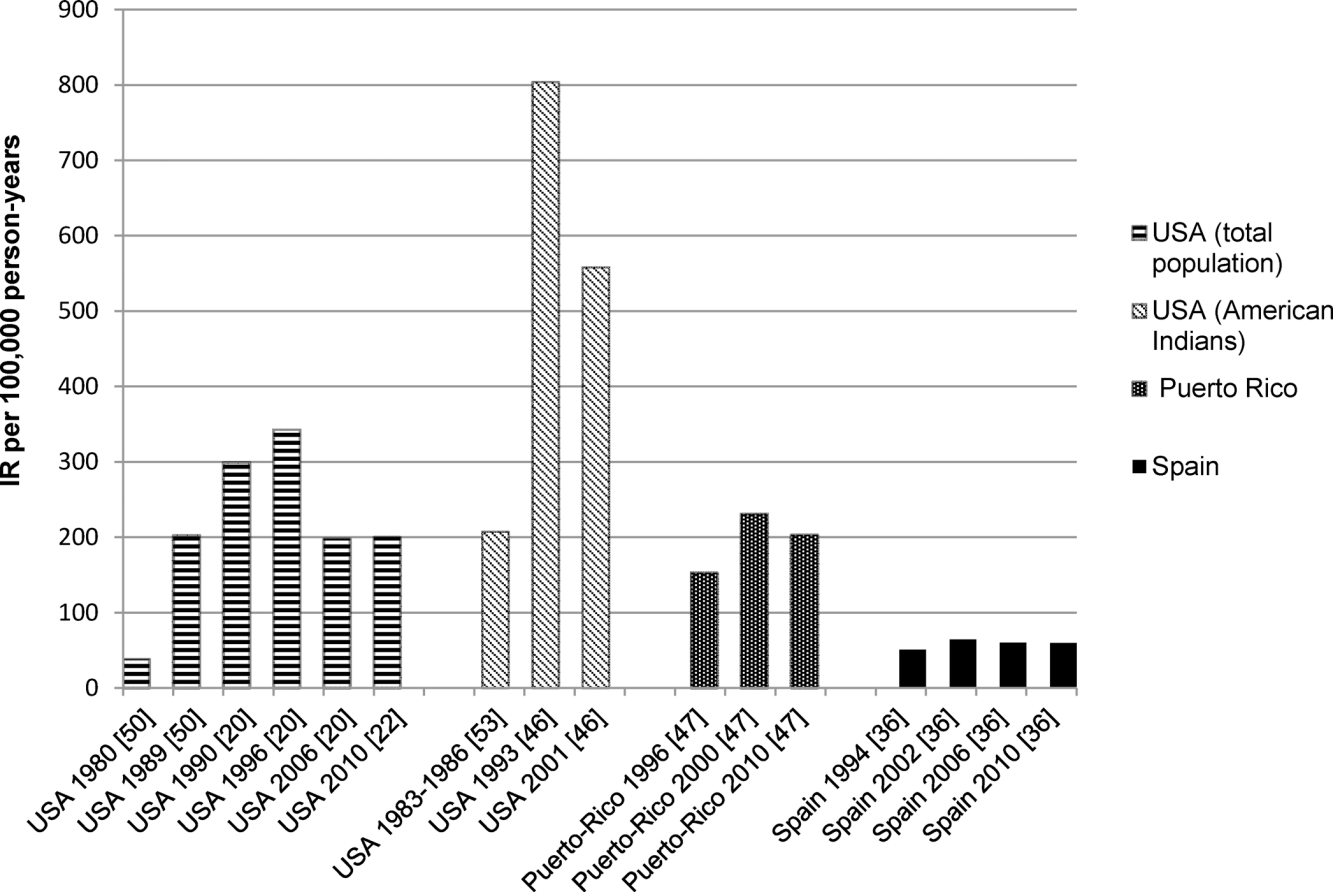
Time trend of age-adjusted incidence rates of ESRD due to diabetic nephropathy. ESRD, end-stage renal disease; IR, incidence rate. Confidence intervals (CI) were not shown as not all studies reported incidence rates of ESRD with the corresponding 95% CIs.

1. Studies which analyzed cumulative incidence of ESRD due to all causes among individuals with type 1 diabetes found that the risk of ESRD was lower for patients whose diagnosis had been established in more recent years [38–40]. 2. In contrast, studies analyzing incidence rates of ESRD due to all causes have demonstrated no clear time trend: no significant time trends were observed in the German study [21], while a Canadian study [3] demonstrated a slight decline from 1994 to 2001. However, the time span in these studies was relatively short, and no differentiation between diabetes types was made. 3. Studies which analyzed incidence rates of ESRD due to DN reported that in the USA the highest level of incidence was reached in 1995/1996 with following reduction. [20, 22, 25, 35, 46, 49–51]. On the Spanish mainland, a similar tendency was observed with an increasing IR up to 2002, followed by a slight decrease [36]. A compatible time trend was described in Puerto Rico [47] (Fig 3). By contrast, the IR of ESRD on the Canary Islands continuously increased even thereafter [55]. The increase of IR ESRD among diabetic patients during the 1980s and early 1990s might be explained by improvements especially in the treatment of cardiovascular disease among patients with DM, resulting in longer survival but followed by a high risk at the same time for another later complication such as DN and its final stage ESRD. However, this hypothesis cannot be discussed in more detail, since only few studies considered cardiovascular comorbidities, and no study investigated interactions between diabetes and comorbidities in the incidence of ESRD. Another explanation might be the increasing number of dialysis facilities and greater access opportunities for diabetic patients.

The described decline or stabilization of IR ESRD due to DN since the mid 1990’s [20, 22, 36, 46] may be regarded as a success in the treatment of patients with DM. However, the results described by Burrows were critically discussed [62]. The problem is that among diabetic patients the IR of ESRD due to DN—regardless of any other chronic illness such as hypertension or renal disease with causes other than diabetes—may be difficult to evaluate. Furthermore, this study did not take the type of diabetes into account. It should be noted that type 1 diabetes and type 2 diabetes are diseases with different pathogenic paths and it is therefore not surprising that dissimilar time trends of ESRD incidence rates between type 1 and type 2 DM were found. A recent French study conducted between 2007 and 2011 which evaluated the incidence of ESRD among diabetic individuals found a decrease in the IR of ESRD among patients with type 1 DM over time (about 10% annually), while the IR increased among patients with type 2 DM (about 7% annually) up to 2009 and stabilized thereafter [61]. A similar tendency was shown in a study analyzing IRs of ESRD among persons with diabetes in Australia and New Zealand between 1991 and 2005, revealing an increase of the IR of ESRD among patients with type 2 DM and a small decrease among patients with type 1 DM [63]. Likewise, Steward et al. reported a compatible trend with a reduction of ESRD incidence rates among diabetic individuals between 1998 and 2001 of 7.8% per year among patients with type 1 DM, and an increment of 9.9% in the same time among patients with type 2 DM [64]. However, these studies solely reported IRs of ESRD within the general population and consequently did not account for the worldwide increase in diabetes prevalence. Therefore, it would be preferable to estimate IRs of ESRD in diabetic populations stratified by diabetes type, taking into account both ESRD due to all causes and due to DN [62].

### Strengths and Limitations

The selection of studies for this systematic review was based on a systematic search approach with clearly determined search strategies. Two independent reviewers screened the articles and performed the data extraction. We included only those studies reporting IR of ESRD within the population at risk, i.e. the diabetic population. The advantage of this method over IR of ESRD within the general population is that the results are not influenced by changes in the prevalence of diabetes. Moreover, we analyzed incidences of ESRD in the diabetic population in separate groups according to epidemiological parameters (IR or CumI), definition of ESRD and study design (study characteristics). This approach allows limited comparison of the studies despite a high degree of heterogeneity. Our review also has some limitations. Although seven databases were searched, we cannot rule out having missed relevant studies, also due to publication bias. Studies which were published in languages other than English were not included. Most studies reporting on IRs of ESRD among patients with DM within the diabetic population were conducted in economically developed areas such as the USA, Europe, Canada and Japan and thus do not represent a worldwide perspective.

The change in the diagnostic criteria for diabetes from 140 mg/dl (7.8 mmol/l) to 126 mg/dl (7.0 mmol/l) in the fasting plasma glucose level in 1997 [65] led to an increase of the diabetic population due to the inclusion of less severe stages of the disease, and this must be taken into consideration when interpreting the results.

## Conclusion

The review conducted demonstrates the considerable variation in incidence of ESRD among the diabetic population. Most studies found a higher incidence of ESRD among male diabetic patients, which was particularly true for the white population. Black, Hispanic, Asian and American Indians/First Nations people have a higher risk of ESRD than white individuals. The incidence of ESRD was substantially higher in the USA than in Europe and Canada. As expected, the incidence of ESRD in the diabetic population was higher than in the non-diabetic population. Owing to the high degree of heterogeneity regarding the causes of ESRD, diabetic populations and types of diabetes, it was difficult to compare the studies. The results concerning time trends were inconsistent: studies reporting incidence of ESRD due to all causes revealed no clear time trend, whereas studies reporting incidence of ESRD due to DN have shown a reduction since the early 2000s.

We recommend that new studies analyzing the incidence of ESRD in the diabetic population should use more consistent definitions concerning the determination of ESRD and population at risk.

## Supporting Information

S1 Table PRISMA Checklist. Preferred Reporting Items for Systematic Reviews and Meta-Analyses (PRISMA) statement checklist.
(DOC)

S1 Text Search strategies. (DOCX)
