# Supplementary material for: The Incidence of End-Stage Renal Disease in the Diabetic (Compared to the Non-Diabetic) Population: A Systematic Review
Source: PLoS One. 2016 Jan 26;11(1):e0147329. doi: 10.1371/journal.pone.0147329 (PMC4727808; doi:10.1371/journal.pone.0147329)
Supplement: S1 Text — (DOCX) [file pone.0147329.s002.docx]

# Supporting Information S1 Text

# Search strategies

Report of search strategies as conducted in February 2013. All strategies have been updated in January 2015.

#### 1 MEDLINE

Database: MEDLINE (PubMed, NLM)

Search period: unlimited

Date of search: 23.02.2013

| **Step** | **Hits** | **Search** |
| --- | --- | --- |
| #1 | 2444 | "DIABETIC NEPHROPATHIES/EPIDEMIOLOGY"[MESH] |
| #2 | 18054 | "DIABETIC NEPHROPATHIES"[MESH] |
| #3 | 73741 | ((("KIDNEY DISEASES, CYSTIC"[MESH]) OR "NEPHRITIS"[MESH]) OR "NEPHROSCLEROSIS"[MESH]) |
| #4 | 12230 | kimmelstiel-wilson [tiab] OR kimmelstiehl-wilson [tiab] OR diabetic glomerulosclerosis [tiab] OR intercapillary glomerulonephritis [tiab] OR diabetic glomerulohyalinosis [tiab] OR diabetic nephropathy [tiab] OR diabetic nephropathies [tiab] OR diabetic kidney disease [tiab] OR diabetic glomerulosclerosis [tiab] OR kimmelstiel wilson [tiab] OR nodular glomerulosclerosis [tiab] |
| #5 | 19819 | "EPIDEMIOLOGY"[MESH] |
| #6 | 472425 | epidemiolog* [ti] OR prevalence [ti] OR incidence [ti] OR frequency [ti] OR population survey [tiab] OR survey data [tiab] OR administrat* data [tiab] OR occurence [ti] OR morbidity [ti] OR mortality [ti] OR population data [tiab] OR population-based [tiab] OR administrative data [tiab] OR community data [tiab] OR community-based [tiab] |
| #7 | 22151 | #2 OR #4 |
| #8 | 490518 | #5 OR #6 |
| #9 | 201844 | (("DIABETES MELLITUS"[MESH:NOEXP]) OR "DIABETES MELLITUS, TYPE 1"[MESH]) OR "DIABETES MELLITUS, TYPE 2"[MESH] |
| #10 | 210616 | diabetes [ti] OR diabetic [ti] |
| #11 | 280446 | #9 OR #10 |
| #12 | 919 | #7 AND #8 |
| #13 | 2958 | #1 OR #12 |
| #14 | 183070 | (("DIALYSIS"[MESH]) OR "RENAL REPLACEMENT THERAPY"[MESH]) OR "KIDNEYS, ARTIFICIAL"[MESH] |
| #15 | 127539 | haemodialysis [tiab] OR hemodialysis [tiab] OR dialysis [tiab] OR hemofiltration [tiab] OR hemodiafiltration [tiab] OR renal dialysis [tiab] OR peritoneal dialysis [tiab] OR capd [tiab] OR kidney transplantation [tiab] OR pancreas-kidney-transplantation [tiab] OR renal replacement therapy [tiab] OR kidney transplantation [tiab] OR kidney replacement therapy [tiab] |
| #16 | 219207 | #14 OR #15 |
| #17 | 377 | #8 AND #11 AND #16 |
| #18 | 92248 | terminal renal failure [tiab] OR terminal kidney failure [tiab] OR glomerulonephritis [tiab] OR glomerulosclerosis [tiab] OR nephropathy [tiab] OR nephropathies [tiab] OR chronic pyelonephritis [tiab] OR progressive glomerulonephritis [tiab] OR interstitial nephritis [tiab] OR cystic kidney [tiab] OR polycystic kidney OR cystic kidneys [tiab] OR ESRD [tiab] OR end-stage renal disease [tiab] OR endstage renal disease [tiab] |
| #19 | 128913 | #3 OR #18 |
| #20 | 14815 | #11 AND #19 |
| #21 | 706 | #8 AND #20 |
| #22 | 3400 | #13 OR #21 OR #17 |
| #23 | 395 | #22 AND (epidemiolog* [ti] OR prevalence [ti] OR incidence [ti] OR morbidity [ti] OR mortality [ti]) AND (end stage renal [ti] OR end stage kidney [ti] OR transplantation [ti] OR dialysis [ti] OR dialyses [ti] OR dialytic [ti] OR hemodialysis [ti] OR hemodialytic [ti] OR hemofiltrat* [ti] OR hemodiafiltrat* [ti] OR esrd [ti] OR nephropath* [ti] OR capd [ti] OR pyelonephriti* [ti]) |
| #24 | 63 | (epidemiology [ti] OR epidemiologic [ti] OR epidemiological [ti] OR prevalence [ti] OR incidence [ti]) AND (diabetes[ti] OR diabetic [ti]) AND (end stage renal [ti] OR end stage kidney [ti] OR dialysis [ti] OR dialyses [ti] OR dialytic [ti] OR hemodialysis [ti] OR hemodialytic [ti] OR hemofiltrat* [ti] OR hemodiafiltrat* [ti] OR esrd [ti] OR capd [ti] OR pyelonephriti* [ti]) |
| #25 | 286 | #22 AND (survey [ti] OR administrat* [ti] OR population [ti] OR population-based [ti] OR community [ti] OR community-based [ti] OR register [ti] OR registers [ti] OR registry [ti] OR registries [ti] OR registered [ti] OR nationwide [ti] OR national [ti] OR international [ti] OR worldwide [ti]) |
| #26 | 245 | #22 AND (epidemiolog* [tiab] OR prevalence [tiab] OR incidence [tiab] OR morbidity [tiab] OR mortality [tiab]) AND (end stage renal [ti] OR end stage kidney [ti] OR transplantation [ti] OR dialysis [ti] OR dialyses [ti] OR dialytic [ti] OR hemodialysis [ti] OR hemodialytic [ti] OR hemofiltrat* [ti] OR hemodiafiltrat* [ti] OR esrd [ti] OR capd [ti] OR pyelonephriti* [ti]) AND (survey [tiab] OR administrat* [tiab] OR population [tiab] OR population-based [tiab] OR community [tiab] OR community-based [tiab] OR register [tiab] OR registers [tiab] OR registry [tiab] OR registries [tiab] OR registered [tiab] OR nationwide [tiab] OR national [tiab] OR worldwide [tiab] OR international [tiab]) |
| #27 | 747 | #23 OR #24 OR #25 OR #26 |
| #28 | 49 | (epidemiolog* [ti] OR prevalence [ti] OR incidence [ti] OR morbidity [ti] OR mortality [ti]) AND (survey [ti] OR administrat* [ti] OR population [ti] OR population-based [ti] OR community [ti] OR community-based [ti] OR register [ti] OR registers [ti] OR registry [ti] OR registries [ti] OR registered [ti] OR nationwide [ti] OR national [ti] OR international [ti] OR worldwide [ti]) AND (end stage renal [ti] OR end stage kidney [ti] OR terminal renal [ti] OR terminal kidney[ti] OR dialysis [ti] OR dialyses [ti] OR dialytic [ti] OR hemodialysis [ti] OR hemodialytic [ti] OR hemofiltrat* [ti] OR hemodiafiltrat* [ti] OR esrd [ti] OR capd [ti]) AND (diabetes [tiab] OR diabetic [tiab]) |
| #29 | 776 | #27 OR #28 |

#### 2 EMBASE, Journals@OVID

Database: MEDLINE (1946-2013), EMBASE (1974-2013), Journals@OVID (OVID)

Search period: unlimited

Date of search: 23.02.2013

| **Step** | **Hits** | **Search** |
| --- | --- | --- |
| 1 | 1364 | *DIABETIC NEPHROPATHIES/ep [EPIDEMIOLOGY] |
| 2 | 26143 | *DIABETIC NEPHROPATHIES/ |
| 3 | 23666 | *KIDNEY DISEASES, CYSTIC/ OR *NEPHRITIS/ OR *NEPHROSCLEROSIS/ |
| 4 | 31257 | (kimmelstiel-wilson OR kimmelstiehl-wilson OR diabetic glomerulosclerosis OR intercapillary glomerulonephritis OR diabetic glomerulohyalinosis OR diabetic nephropathy OR diabetic nephropathies OR diabetic kidney disease OR diabetic glomerulosclerosis OR kimmelstiel wilson OR nodular glomerulosclerosis).ti,ab. |
| 5 | 43618 | *EPIDEMIOLOGY/ |
| 6 | 1206998 | (epidemiolog* OR prevalence OR incidence OR frequency OR occurence OR morbidity OR mortality).ti. OR (population survey OR survey data OR administrat* data OR population data OR population-based OR administrative data OR community data OR community-based).ti,ab. |
| 7 | 41290 | 2 OR 4 |
| 8 | 1230821 | 5 OR 6 |
| 9 | 430664 | *DIABETES MELLITUS/ OR *DIABETES MELLITUS, TYPE 1/ OR *DIABETES MELLITUS, TYPE 2/ |
| 10 | 567093 | (diabetes OR diabetic).m_titl. |
| 11 | 667874 | 9 OR 10 |
| 12 | 219645 | (terminal renal failure OR terminal kidney failure OR glomerulonephritis OR glomerulosclerosis OR nephropathy OR nephropathies OR chronic pyelonephritis OR progressive glomerulonephritis OR interstitial nephritis OR cystic kidney OR polycystic kidney OR cystic kidneys OR esrd OR end-stage renal disease OR endstage renal disease).ti,ab. |
| 13 | 1726 | 7 AND 8 |
| 14 | 2678 | 1 OR 13 |
| 15 | 235949 | 3 OR 12 |
| 16 | 35146 | 11 AND 15 |
| 17 | 2047 | 8 AND 16 |
| 18 | 10085 | *DIALYSIS/ |
| 19 | 163956 | *RENAL REPLACEMENT THERAPY/ OR *DIALYSIS/ OR *HEMOFILTRATION/ OR *KIDNEY TRANSPLANTATION/ |
| 20 | 330162 | (haemodialysis OR hemodialysis OR dialysis OR hemofiltration OR hemodiafiltration OR renal dialysis OR peritoneal dialysis OR capd OR kidney transplantation OR pancreas-kidney-transplantation OR renal replacement therapy OR kidney transplantation OR kidney replacement therapy).ti,ab. |
| 21 | 430028 | 18 OR 19 OR 20 |
| 22 | 802 | 8 AND 11 AND 21 |
| 23 | 4078 | 14 OR 17 OR 22 |
| 24 | 3111 | 6 AND 23 |
| 25 | 3017 | 24 AND ((population survey OR survey data OR administrat* data OR population data OR population-based OR administrative data OR community data OR community-based OR register OR registers OR registry OR registries OR registered OR observational stud* OR national cohort OR nationwide cohort).ti,ab. OR (national OR nationwide OR international OR epidemiolog* OR prevalence OR incidence OR survey OR morbidity OR mortality OR community).ti.) |
| 26 | 2580 | 24 AND (epidemiolog* OR prevalence OR incidence OR morbidity OR mortality).ti. |
| 27 | 1073 | 24 AND (epidemiolog* OR prevalence OR incidence OR morbidity OR mortality).ti. AND (terminal renal OR terminal kidney OR end stage renal OR end stage kidney OR transplantation OR dialysis OR dialyses OR dialytic OR hemodialysis OR hemodialytic OR hemofiltrat* OR hemodiafiltrat* OR esrd OR nephro* OR nephri* OR glomerulo* OR capd OR pyelonephriti*).ti. |
| 28 | 406 | 24 AND (survey OR administrat* OR population OR population-based OR community OR community-based OR register OR registers OR registry OR registries OR registered OR nationwide OR national OR international OR worldwide).ti. |
| 29 | 489 | 24 AND ((epidemiolog* OR prevalence OR incidence OR morbidity OR mortality).ti,ab. AND (stage renal OR end stage kidney OR terminal renal OR terminal kidney OR transplantation OR dialysis OR dialyses OR dialytic OR hemodialysis OR hemodialytic OR hemofiltrat* OR hemodiafiltrat* OR esrd OR nephro* OR nephri* OR glomerulo* OR capd OR pyelonephriti*).ti. AND (survey OR administrat* OR population OR population-based OR community OR community-based OR register OR registers OR registry OR registries OR registered OR nationwide OR national OR worldwide OR international).ti,ab.) |
| 30 | 490 | ((epidemiology OR epidemiologic OR epidemiological OR prevalence OR incidence) AND (diabetes OR diabetic) AND (stage renal OR end stage kidney OR terminal renal OR terminal kidney OR dialysis OR dialyses OR dialytic OR hemodialysis OR hemodialytic OR hemofiltrat* OR hemodiafiltrat* OR esrd OR nephro* OR nephri* OR glomerulo* OR capd OR pyelonephriti*)).m_titl. |
| 31 | 116 | ((epidemiolog* OR prevalence OR incidence OR morbidity OR mortality) AND (survey OR administrat* OR population OR population-based OR community OR community-based OR register OR registers OR registry OR registries OR registered OR nationwide OR national OR international OR worldwide) AND (end stage renal OR end stage kidney OR terminal renal OR terminal kidney OR dialysis OR dialyses OR dialytic OR hemodialysis OR hemodialytic OR hemofiltrat* OR hemodiafiltrat* OR esrd OR capd)).ti. AND (diabetes OR diabetic).ti,ab. |
| 32 | 1524 | 27 OR 28 OR 29 OR 30 OR 31 |
| 33 | 1497 | 32 not (hospital OR inpatient OR department OR clinic).ti. |
| 34 | 817 | remove duplicates from 33  MEDLINE: 568  EMBASE: 184  Journals@Ovid: 65 |

#### 3 Web of Knowledge

Database: Web of Knowledge (Thomson Reuters)

Search period: unlimited

Date of search: 23.02.2013

| **Step** | **Hits** | **Search** |
| --- | --- | --- |
| 1 | 314 | Title=((epidemiology OR epidemiologic OR epidemiological OR prevalence OR incidence)) AND Title=((diabetes OR diabetic)) AND Title=((terminal renal OR terminal kidney OR end stage renal OR end stage kidney OR dialysis OR dialyses OR dialytic OR hemodialysis OR hemodialytic OR hemofiltrat* OR hemodiafiltrat* OR esrd OR nephro* OR nephri* OR glomerulo* OR capd OR pyelonephriti*)) Timespan=All Years Lemmatization=On |

#### 4 ScienceDirect

Database: ScienceDirect (Elsevier, Segmente “Medicine and Dentistry”, “Neuroscience”, “Nursing and Health professions”)

Search period: unlimited

Date of search: 23.02.2013

| **Step** | **Hits** | **Search** |
| --- | --- | --- |
| 1 | 3 | TITLE((epidemiology OR epidemiologic OR epidemiological OR prevalence OR incidence)) and TITLE-ABSTR-KEY((terminal renal OR terminal kidney OR end stage renal OR end stage kidney OR dialysis OR dialyses OR dialytic OR hemodialysis OR hemodialytic OR hemofiltrat* OR hemodiafiltrat* OR esrd OR nephro* OR nephri* OR glomerulo* OR capd OR pyelonephriti*)) |

#### 5 CCMed, Deutsches Ärzteblatt

Database: CCMed, Deutsches Ärzteblatt (DIMDI)

Search period: unlimited

Date of search: 23.02.2013

| **Step** | **Hits** | **Search** |
| --- | --- | --- |
| 1 | 128 | (FT=(epidemiolog? ; prevalence ; prävalenz? ; incidence ; inzidenz? ) AND FT=(diabetes ; diabeti? )) AND FT=(end stage renal ; end stage kidney? ; terminale nieren? ; dialysis ; dialyses ; dialytic ; hemodialysis ; hemodialytic ; hemofiltrat? ; hemodiafiltrat? ; esrd ; nephro? ; nephri? ; glomerulo? ; capd ; pyelonephriti? ) |

Update: Time period up to 3^th^ of January 2015

#### 1 MEDLINE

Database: MEDLINE (PubMed, NLM)

Search period: 2013-2015

Date of search: 03.01.2015

| **Step** | **Hits** | **Search** |
| --- | --- | --- |
| #1 | 2685 | "DIABETIC NEPHROPATHIES/EPIDEMIOLOGY"[MESH] |
| #2 | 19606 | "DIABETIC NEPHROPATHIES"[MESH] |
| #3 | 77057 | ((("KIDNEY DISEASES, CYSTIC"[MESH]) OR "NEPHRITIS"[MESH]) OR "NEPHROSCLEROSIS"[MESH]) |
| #4 | 14037 | kimmelstiel-wilson [tiab] OR kimmelstiehl-wilson [tiab] OR diabetic glomerulosclerosis [tiab] OR intercapillary glomerulonephritis [tiab] OR diabetic glomerulohyalinosis [tiab] OR diabetic nephropathy [tiab] OR diabetic nephropathies [tiab] OR diabetic kidney disease [tiab] OR diabetic glomerulosclerosis [tiab] OR kimmelstiel wilson [tiab] OR nodular glomerulosclerosis [tiab] |
| #5 | 21575 | "EPIDEMIOLOGY"[MESH] |
| #6 | 546067 | epidemiolog* [ti] OR prevalence [ti] OR incidence [ti] OR frequency [ti] OR population survey [tiab] OR survey data [tiab] OR administrat* data [tiab] OR occurence [ti] OR morbidity [ti] OR mortality [ti] OR population data [tiab] OR population-based [tiab] OR administrative data [tiab] OR community data [tiab] OR community-based [tiab] |
| #7 | 24552 | #2 OR #4 |
| #8 | 565690 | #5 OR #6 |
| #9 | 222637 | (("DIABETES MELLITUS"[MESH:NOEXP]) OR "DIABETES MELLITUS, TYPE 1"[MESH]) OR "DIABETES MELLITUS, TYPE 2"[MESH] |
| #10 | 237521 | diabetes [ti] OR diabetic [ti] |
| #11 | 314274 | #9 OR #10 |
| #12 | 1049 | #7 AND #8 |
| #13 | 3289 | #1 OR #12 |
| #14 | 194471 | (("DIALYSIS"[MESH]) OR "RENAL REPLACEMENT THERAPY"[MESH]) OR "KIDNEYS, ARTIFICIAL"[MESH] |
| #15 | 140058 | haemodialysis [tiab] OR hemodialysis [tiab] OR dialysis [tiab] OR hemofiltration [tiab] OR hemodiafiltration [tiab] OR renal dialysis [tiab] OR peritoneal dialysis [tiab] OR capd [tiab] OR kidney transplantation [tiab] OR pancreas-kidney-transplantation [tiab] OR renal replacement therapy [tiab] OR kidney transplantation [tiab] OR kidney replacement therapy [tiab] |
| #16 | 235946 | #14 OR #15 |
| #17 | 448 | #8 AND #11 AND #16 |
| #18 | 101521 | terminal renal failure [tiab] OR terminal kidney failure [tiab] OR glomerulonephritis [tiab] OR glomerulosclerosis [tiab] OR nephropathy [tiab] OR nephropathies [tiab] OR chronic pyelonephritis [tiab] OR progressive glomerulonephritis [tiab] OR interstitial nephritis [tiab] OR cystic kidney [tiab] OR polycystic kidney OR cystic kidneys [tiab] OR ESRD [tiab] OR end-stage renal disease [tiab] OR endstage renal disease [tiab] |
| #19 | 139592 | #3 OR #18 |
| #20 | 16684 | #11 AND #19 |
| #21 | 832 | #8 AND #20 |
| #22 | 3830 | #13 OR #21 OR #17 |
| #23 | 450 | #22 AND (epidemiolog* [ti] OR prevalence [ti] OR incidence [ti] OR morbidity [ti] OR mortality [ti]) AND (end stage renal [ti] OR end stage kidney [ti] OR transplantation [ti] OR dialysis [ti] OR dialyses [ti] OR dialytic [ti] OR hemodialysis [ti] OR hemodialytic [ti] OR hemofiltrat* [ti] OR hemodiafiltrat* [ti] OR esrd [ti] OR nephropath* [ti] OR capd [ti] OR pyelonephriti* [ti]) |
| #24 | 70 | (epidemiology [ti] OR epidemiologic [ti] OR epidemiological [ti] OR prevalence [ti] OR incidence [ti]) AND (diabetes[ti] OR diabetic [ti]) AND (end stage renal [ti] OR end stage kidney [ti] OR dialysis [ti] OR dialyses [ti] OR dialytic [ti] OR hemodialysis [ti] OR hemodialytic [ti] OR hemofiltrat* [ti] OR hemodiafiltrat* [ti] OR esrd [ti] OR capd [ti] OR pyelonephriti* [ti]) |
| #25 | 346 | #22 AND (survey [ti] OR administrat* [ti] OR population [ti] OR population-based [ti] OR community [ti] OR community-based [ti] OR register [ti] OR registers [ti] OR registry [ti] OR registries [ti] OR registered [ti] OR nationwide [ti] OR national [ti] OR international [ti] OR worldwide [ti]) |
| #26 | 274 | #22 AND (epidemiolog* [tiab] OR prevalence [tiab] OR incidence [tiab] OR morbidity [tiab] OR mortality [tiab]) AND (end stage renal [ti] OR end stage kidney [ti] OR transplantation [ti] OR dialysis [ti] OR dialyses [ti] OR dialytic [ti] OR hemodialysis [ti] OR hemodialytic [ti] OR hemofiltrat* [ti] OR hemodiafiltrat* [ti] OR esrd [ti] OR capd [ti] OR pyelonephriti* [ti]) AND (survey [tiab] OR administrat* [tiab] OR population [tiab] OR population-based [tiab] OR community [tiab] OR community-based [tiab] OR register [tiab] OR registers [tiab] OR registry [tiab] OR registries [tiab] OR registered [tiab] OR nationwide [tiab] OR national [tiab] OR worldwide [tiab] OR international [tiab]) |
| #27 | 866 | #23 OR #24 OR #25 OR #26 |
| #28 | 62 | (epidemiolog* [ti] OR prevalence [ti] OR incidence [ti] OR morbidity [ti] OR mortality [ti]) AND (survey [ti] OR administrat* [ti] OR population [ti] OR population-based [ti] OR community [ti] OR community-based [ti] OR register [ti] OR registers [ti] OR registry [ti] OR registries [ti] OR registered [ti] OR nationwide [ti] OR national [ti] OR international [ti] OR worldwide [ti]) AND (end stage renal [ti] OR end stage kidney [ti] OR terminal renal [ti] OR terminal kidney[ti] OR dialysis [ti] OR dialyses [ti] OR dialytic [ti] OR hemodialysis [ti] OR hemodialytic [ti] OR hemofiltrat* [ti] OR hemodiafiltrat* [ti] OR esrd [ti] OR capd [ti]) AND (diabetes [tiab] OR diabetic [tiab]) |
| #29 | 903 | #27 OR #28 |
| #30 | 110 | #29 AND (2013:2015 [edat]) |
| #31 | 113 | #29 AND (2013:2015 [crdat]) |
| #32 | 119 | #27 OR #28 Filters: Publication date from 2013/01/01 to 2015/12/31 |
| #33 | 123 | #30 OR #31 OR #32 |

#### 2 EMBASE, Journals@OVID

Database: MEDLINE (1946-2015), EMBASE (1974-2015), Journals@OVID (OVID)

Search period: 2013-2015

Date of search: 03.01.2015

| **Step** | **Hits** | **Search** |
| --- | --- | --- |
| 1 | 1453 | *DIABETIC NEPHROPATHIES/ep [EPIDEMIOLOGY] |
| 2 | 28865 | *DIABETIC NEPHROPATHIES/ |
| 3 | 24553 | *KIDNEY DISEASES, CYSTIC/ OR *NEPHRITIS/ OR *NEPHROSCLEROSIS/ |
| 4 | 36451 | (kimmelstiel-wilson OR kimmelstiehl-wilson OR diabetic glomerulosclerosis OR intercapillary glomerulonephritis OR diabetic glomerulohyalinosis OR diabetic nephropathy OR diabetic nephropathies OR diabetic kidney disease OR diabetic glomerulosclerosis OR kimmelstiel wilson OR nodular glomerulosclerosis).ti,ab. |
| 5 | 51625 | *EPIDEMIOLOGY/ |
| 6 | 1399938 | (epidemiolog* OR prevalence OR incidence OR frequency OR occurence OR morbidity OR mortality).ti. OR (population survey OR survey data OR administrat* data OR population data OR population-based OR administrative data OR community data OR community-based).ti,ab. |
| 7 | 47028 | 2 OR 4 |
| 8 | 1428659 | 5 OR 6 |
| 9 | 492850 | *DIABETES MELLITUS/ OR *DIABETES MELLITUS, TYPE 1/ OR *DIABETES MELLITUS, TYPE 2/ |
| 10 | 654870 | (diabetes OR diabetic).m_titl. |
| 11 | 769140 | 9 OR 10 |
| 12 | 246931 | (terminal renal failure OR terminal kidney failure OR glomerulonephritis OR glomerulosclerosis OR nephropathy OR nephropathies OR chronic pyelonephritis OR progressive glomerulonephritis OR interstitial nephritis OR cystic kidney OR polycystic kidney OR cystic kidneys OR esrd OR end-stage renal disease OR endstage renal disease).ti,ab. |
| 13 | 1971 | 7 AND 8 |
| 14 | 2980 | 1 OR 13 |
| 15 | 263603 | 3 OR 12 |
| 16 | 40370 | 11 AND 15 |
| 17 | 2401 | 8 AND 16 |
| 18 | 11259 | *DIALYSIS/ |
| 19 | 172110 | *RENAL REPLACEMENT THERAPY/ OR *DIALYSIS/ OR *HEMOFILTRATION/ OR *KIDNEY TRANSPLANTATION/ |
| 20 | 371308 | (haemodialysis OR hemodialysis OR dialysis OR hemofiltration OR hemodiafiltration OR renal dialysis OR peritoneal dialysis OR capd OR kidney transplantation OR pancreas-kidney-transplantation OR renal replacement therapy OR kidney transplantation OR kidney replacement therapy).ti,ab. |
| 21 | 474596 | 18 OR 19 OR 20 |
| 22 | 980 | 8 AND 11 AND 21 |
| 23 | 4703 | 14 OR 17 OR 22 |
| 24 | 3675 | 6 AND 23 |
| 25 | 3566 | 24 AND ((population survey OR survey data OR administrat* data OR population data OR population-based OR administrative data OR community data OR community-based OR register OR registers OR registry OR registries OR registered OR observational stud* OR national cohort OR nationwide cohort).ti,ab. OR (national OR nationwide OR international OR epidemiolog* OR prevalence OR incidence OR survey OR morbidity OR mortality OR community).ti.) |
| 26 | 3022 | 24 AND (epidemiolog* OR prevalence OR incidence OR morbidity OR mortality).ti. |
| 27 | 1242 | 24 AND (epidemiolog* OR prevalence OR incidence OR morbidity OR mortality).ti. AND (terminal renal OR terminal kidney OR end stage renal OR end stage kidney OR transplantation OR dialysis OR dialyses OR dialytic OR hemodialysis OR hemodialytic OR hemofiltrat* OR hemodiafiltrat* OR esrd OR nephro* OR nephri* OR glomerulo* OR capd OR pyelonephriti*).ti. |
| 28 | 498 | 24 AND (survey OR administrat* OR population OR population-based OR community OR community-based OR register OR registers OR registry OR registries OR registered OR nationwide OR national OR international OR worldwide).ti. |
| 29 | 570 | 24 AND ((epidemiolog* OR prevalence OR incidence OR morbidity OR mortality).ti,ab. AND (stage renal OR end stage kidney OR terminal renal OR terminal kidney OR transplantation OR dialysis OR dialyses OR dialytic OR hemodialysis OR hemodialytic OR hemofiltrat* OR hemodiafiltrat* OR esrd OR nephro* OR nephri* OR glomerulo* OR capd OR pyelonephriti*).ti. AND (survey OR administrat* OR population OR population-based OR community OR community-based OR register OR registers OR registry OR registries OR registered OR nationwide OR national OR worldwide OR international).ti,ab.) |
| 30 | 551 | ((epidemiology OR epidemiologic OR epidemiological OR prevalence OR incidence) AND (diabetes OR diabetic) AND (stage renal OR end stage kidney OR terminal renal OR terminal kidney OR dialysis OR dialyses OR dialytic OR hemodialysis OR hemodialytic OR hemofiltrat* OR hemodiafiltrat* OR esrd OR nephro* OR nephri* OR glomerulo* OR capd OR pyelonephriti*)).m_titl. |
| 31 | 160 | ((epidemiolog* OR prevalence OR incidence OR morbidity OR mortality) AND (survey OR administrat* OR population OR population-based OR community OR community-based OR register OR registers OR registry OR registries OR registered OR nationwide OR national OR international OR worldwide) AND (end stage renal OR end stage kidney OR terminal renal OR terminal kidney OR dialysis OR dialyses OR dialytic OR hemodialysis OR hemodialytic OR hemofiltrat* OR hemodiafiltrat* OR esrd OR capd)).ti. AND (diabetes OR diabetic).ti,ab. |
| 32 | 1796 | 27 OR 28 OR 29 OR 30 OR 31 |
| 33 | 1006 | remove duplicates from 32 |
| 34 | 156 | limit 33 to yr="2013 – 2015  MEDLINE: 22  EMBASE: 125  Journals@Ovid: 9 |
| 35 | 349 | limit 33 to em="201302-201501"  MEDLINE: 111  EMBASE: 160  Journals@Ovid: 78 |
|  |  | exportiert: 78 + 125 + 160 |

#### 3 Web of Knowledge

Datebase: Web of Knowledge (Thomson Reuters)

Search period: 2013-2015

Date of search: 03.01.2015

| **Step** | **Hits** | **Search** |
| --- | --- | --- |
| 1 | 18 | TI=((epidemiology OR epidemiologic OR epidemiological OR prevalence OR incidence)) AND TI=((diabetes OR diabetic)) AND TI=((terminal renal OR terminal kidney OR end stage renal OR end stage kidney OR dialysis OR dialyses OR dialytic OR hemodialysis OR hemodialytic OR hemofiltrat* OR hemodiafiltrat* OR esrd OR nephro* OR nephri* OR glomerulo* OR capd OR pyelonephriti*)) Timespan=2013-2015 Lemmatization=On |

#### 4 ScienceDirect

Datebase: ScienceDirect (Elsevier, Segmente “Medicine and Dentistry”, “Neuroscience”, “Nursing and Health professions”)

Search period: 2013-2015

Date of search: 03.01.2015

| **Step** | **Hits** | **Search** |
| --- | --- | --- |
| 1 | 0 | TITLE((epidemiology OR epidemiologic OR epidemiological OR prevalence OR incidence)) and TITLE-ABSTR-KEY((terminal renal OR terminal kidney OR end stage renal OR end stage kidney OR dialysis OR dialyses OR dialytic OR hemodialysis OR hemodialytic OR hemofiltrat* OR hemodiafiltrat* OR esrd OR nephro* OR nephri* OR glomerulo* OR capd OR pyelonephriti*)) |

#### 5 CCMed

Datebase: CCMed (Medpilot, ZBMed)

Search period: 2013-2015

Date of search: 03.01.2015

| **Step** | **Hits** | **Search** |
| --- | --- | --- |
| 1 | 0 | TI=(epidemiolog? OR prevalence OR prävalenz? OR incidence OR inzidenz?) AND TI=(end stage renal OR end stage kidney? OR terminale nieren? OR dialysis OR dialyses OR dialytic OR hemodialysis OR hemodialytic OR hemofiltrat? OR hemodiafiltrat? OR esrd OR nephro? OR nephri? OR glomerulo? OR capd OR pyelonephriti?) |

#### 5 Deutsches Ärzteblatt

Datenbase: Deutsches Ärzteblatt (Ärzte-Verlag)

Search period: 2013-2015

Date of search: 03.01.2015

0 Hits
